# Angiogenic desmoplastic histopathological growth pattern as a prognostic marker of good outcome in patients with colorectal liver metastases

**DOI:** 10.1007/s10456-019-09661-5

**Published:** 2019-01-12

**Authors:** Boris Galjart, Pieter M. H. Nierop, Eric P. van der Stok, Robert R. J. Coebergh van den Braak, Diederik J. Höppener, Sofie Daelemans, Luc Y. Dirix, Cornelis Verhoef, Peter B. Vermeulen, Dirk J. Grünhagen

**Affiliations:** 1 Department of Surgical Oncology, Erasmus MC Cancer Institute, P.O. Box 2040, 3000 CA Rotterdam, The Netherlands; 2 Department of Surgery, Erasmus Medical Centre, Rotterdam, The Netherlands; 3 Medical Biochemistry, Faculty of Pharmaceutical, Biomedical and Veterinary Sciences, University of Antwerp, Antwerp, Belgium; 4 HistoGeneX, Antwerp, Belgium; 5 Translational Cancer Research Unit (GZA Hospitals and University of Antwerp), Antwerp, Belgium

**Keywords:** Histopathological growth pattern, Prognostic factor, Colorectal cancer, Liver metastases, Vessel co-option

## Abstract

**Background:**

In patients with resectable colorectal liver metastases (CRLM), distinct histopathological growth patterns (HGPs) develop at the interface between the tumour and surrounding tissue. The desmoplastic (d) HGP is characterised by angiogenesis and a peripheral fibrotic rim, whereas non-angiogenic HGPs co-opt endogenous sinusoidal hepatic vasculature. Evidence from previous studies has suggested that patients with dHGP in their CRLM have improved prognosis as compared to patients with non-desmoplastic HGPs. However, these studies were relatively small and applied arbitrary cut-off values for the determination of the predominant HGP. We have now investigated the prognostic effect of dHGP in a large cohort of patients with CRLM resected either with or without neoadjuvant chemotherapy.

**Methods:**

All consecutive patients undergoing a first partial hepatectomy for CRLM between 2000 and 2015 at a tertiary referral centre were considered for inclusion. HGPs were assessed in archival H&E stained slides according to recently published international consensus guidelines. The dHGP was defined as desmoplastic growth being present in 100% of the interface between the tumour and surrounding liver.

**Results:**

In total, HGPs in CRLMs from 732 patients were assessed. In the chemo-naive patient cohort (*n* = 367), the dHGP was present in 19% (*n* = 68) and the non-dHGP was present in 81% (*n* = 299) of patients. This dHGP subgroup was independently associated with good overall survival (OS) (HR: 0.39, *p* < 0.001) and progression-free survival (PFS) (HR: 0.54, *p* = 0.001). All patients with any CRLM with a non-dHGP had significantly reduced OS compared to those patients with 100% dHGP, regardless of the proportion of non-dHGP (all *p* values ≤ 0.001). In the neoadjuvantly treated patient cohort (*n* = 365), more patients were found to express dHGP (*n* = 109, 30%) (adjusted odds ratio: 2.71, *p* < 0.001). On univariable analysis, dHGP was associated with better OS (HR 0.66, *p* = 0.009) and PFS (HR 0.67, *p* = 0.002). However, after correction for confounding by means of multivariable analysis no significant association of dHGP with OS (HR 0.92, *p* = 0.623) or PFS (HR 0.76, *p* = 0.065) was seen.

**Conclusions:**

The current study demonstrates that the angiogenic dHGP in CRLM resected from chemo-naive patients acts as a strong, positive prognostic marker, unmatched by any other prognosticator. This observation warrants the evaluation of the clinical utility of HGPs in prospective clinical trials.

**Electronic supplementary material:**

The online version of this article (10.1007/s10456-019-09661-5) contains supplementary material, which is available to authorised users.

## Introduction

As hepatic tumours develop, histopathological growth patterns (HGPs) appear at the interface between the tumour border and surrounding liver parenchyma. Previous studies have suggested that HGPs have the potential to predict both tumour biology and prognosis in patients with colorectal liver metastasis (CRLM). Three primary HGPs have been identified in CRLM: desmoplastic (d), replacement (r) and the pushing (p) pattern [1]. Over time the classification of HGPs has evolved and ultimately resulted in international consensus guidelines. Applying these guidelines made the dHGP and rHGP the most common types and the pHGP fairly uncommon [2].

In addition to the fibrotic reaction (desmoplasia) that surrounds the metastases, one of the predominant features of tumours which exhibit dHGP is angiogenesis. These tumours are characterised by elevated endothelial cell proliferation and regions of increased vessel density called vascular hot spots. The new blood vessels appear leaky and functionally impaired with fibrin deposits in the peri-vascular stroma [3]. This is in contrast to the rHGP, in which angiogenesis does not occur, the proportion of proliferating endothelial cells is very low and there are no obvious effects of VEGFA such as fibrin deposition [3]. In rHGP, vascularisation of the tumours is established by co-opting the existing sinusoidal blood vessels of the liver [3, 4]. As the name implies, cancer cells ‘replace’ the hepatocytes, while the stromal architecture of the liver is maintained.

Tumours with rHGP exhibit features that have been associated with aggressive cancer biology and impaired prognosis, including increased cancer cell motility [4], non-angiogenic growth [4] and reduced infiltration of CD8+ immune cells [5, 6]. Previous studies evaluating the prognostic impact of HGPs suggest that the dHGP is associated with improved prognosis [2, 4, 5, 7–10]. These studies were relatively small and applied arbitrary cut-off values for the determination of the predominant growth pattern. If HGPs are an intrinsic reflection of tumour biology, one could hypothesise that the presence of *any* non-desmoplastic HGP (pHGP and/or rHGP) could be of prognostic value. The current study investigated the association of dHGP with survival in a large cohort of patients undergoing resection of CRLM, and the potential correlation between neoadjuvant chemotherapy and HGPs.

## Methods

### Patient selection and data

All consecutive patients who underwent laparotomy for surgical treatment of CRLM between January 2000 and March 2015 at the Erasmus MC Cancer Institute were considered for inclusion. The Erasmus MC Cancer Institute is a tertiary referral centre for liver surgery. Patients without complete surgical treatment (i.e. resection with or without ablation of all known CRLM) with curative intent were excluded. In addition, patients treated with ablation only were also excluded. Clinicopathological data on primary tumour, CRLM and recurrent metastatic disease were obtained from a prospectively maintained database. HGP assessment was performed retrospectively on H&E stained tissues sections from archival tissue. The current study was performed according to the REMARK guidelines and approved by the medical ethics committee of the Erasmus University Medical Centre Rotterdam (MEC-2016-046) [11].

### Prognosis

The primary objective of this study was to evaluate the association between HGPs and prognosis after first hepatectomy for CRLM. In order to analyse this, HGP data of the first hepatectomy were evaluated (i.e. for the survival analyses recurrent CRLM treated with repeat hepatectomy were not evaluated). Survival was measured as progression-free survival (PFS) and overall survival (OS). The PFS was defined as the time in months between resection of CRLM and diagnosis of progression of disease or death, whichever occurred first. Disease progression was diagnosed by radiological or histological assessment. The OS was defined as the time in months between surgery for CRLM and death.

### Effect of chemotherapy

The secondary objective was to assess the potential association between chemotherapy and the prevalence of HGPs. In order to do so, distribution of HGPs amongst chemo-naive and neoadjuvantly treated patients was compared. Patients who had received any chemotherapy within the 6 months prior to the liver resection were considered neoadjuvantly treated. Patients with a liver recurrence undergoing repeat resection at the Erasmus MC Cancer Institute were identified and subsequently stratified into four distinct treatment groups: chemo-naive at both hepatectomies (−/−), neoadjuvantly treated at the first hepatectomy but chemo-naive at the second (+/−), chemo-naive at the first hepatectomy but neoadjuvantly treated at the second (−/+) and lastly neoadjuvantly treated at both hepatectomies (+/+). Specifically for this secondary objective, the HGPs of these recurrent CRLM were determined as well and the prevalence of HGPs at first and second hepatectomy was compared across these four distinct treatment groups.

### Chemotherapy and follow-up

In accordance with the Dutch national guidelines, (neo)adjuvant chemotherapy is not standard of care for patients with CRLM. Only in case of initially marginally resectable, synchronous and/or multiple (≥ 4) resectable CRLM is neoadjuvant chemotherapy considered. A proportion of patients received neoadjuvant chemotherapy in the referring hospital prior to admission. None of the patients received adjuvant chemotherapy.

Post-operative surveillance is performed for up to 5 years after surgery for CRLM, using thoracoabdominal computed tomography (CT) and carcinoembryonic antigen (CEA)-level measurements every 3–6 months for 3 years and then annually thereafter. After 5 years, further surveillance was performed by the general practitioner. Patients were censored for PFS at date of last follow-up if without disease progression.

### Pathological assessment and description of HGPs

**Fig. 1:**
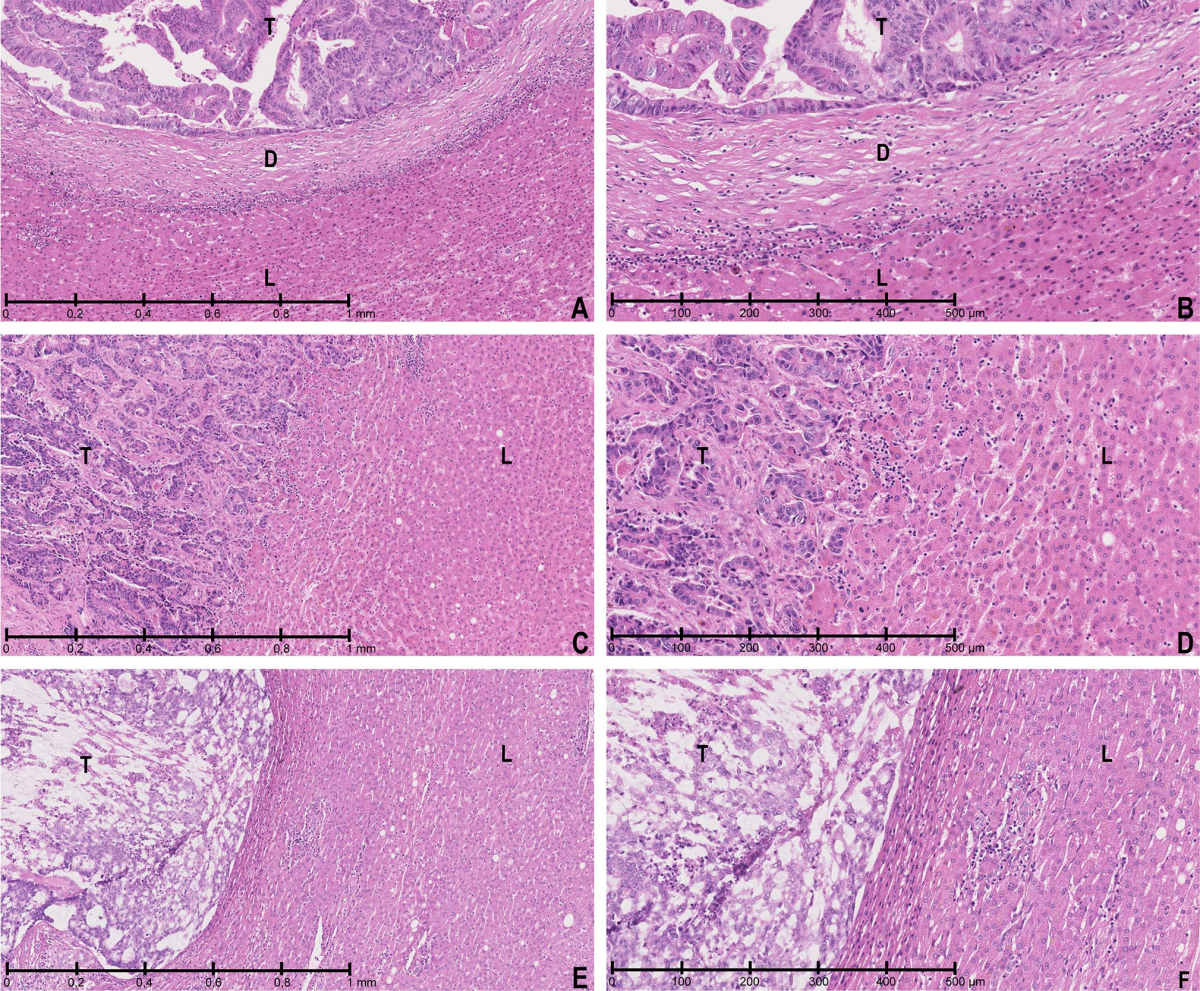
**a–f** Collated HE tissue sections. **a, b** dHGP low and high magnification; **c, d** rHGP low and high magnification; **e, f** pHGP low and high magnification. T: tumour; D: desmoplastic stroma; L: liver parenchyma

HGPs were determined according to the international consensus guidelines of the Liver Metastasis Research Network [2] blinded for patient outcome. HGPs were assessed per patient in all available haematoxylin and eosin (H&E) stained sections from all resected CRLM. In each slide, the interface between tumour border and normal liver parenchyma was evaluated using light microscopy by at least three trained observers (PV, ES, RC, BG, PN, DH). As some CRLM display a combination of HGPs, the entire tumour–liver interface was evaluated for each tissue section. When multiple HGPs were present at the interface, the HGP was scored as a relative proportion of the interface in which each of dHGP, rHGP and/or pHGP occurred. Every fraction of the tumour–liver interface, accounting for 5% or more of the total interface of a metastasis, was taken into account. Average HGP scores were then calculated for each metastasis (in case of multiple slides per CRLM) and patient (in case of multiple CRLM). Tissue sections were considered not suitable for HGP assessment when less than 20% interface was available, when the quality of the H&E tissue section was insufficient for reliable assessment or when viable tumour tissue was absent [2]. Examples of H&E tissue sections with CRLM displaying dHGP, rHGP and pHGP are shown in Fig. 1a–f. In the dHGP, the cancer cells of the metastasis are separated from the liver tissue by a rim of desmoplastic tissue. The metastasis does not mimic the liver architecture and there is no direct contact between cancer cells and hepatocytes (Fig. 1a, b). There is often a dense lymphocytic infiltrate at the interface of the desmoplastic rim and the liver tissue. A ‘ductular reaction’, or proliferation of bile ducts, can sometimes be seen surrounding the desmoplastic metastasis. In the pHGP, the liver cell plates that surround the metastasis are pushed away and compressed (Fig. 1e, f). There is no desmoplastic rim surrounding the metastasis but also no direct contact between cancer cells and hepatocytes within the liver cell plates. As in the dHGP, the metastasis does not mimic the liver architecture. In the rHGP, cancer cells form cell plates that are in continuity with the liver cell plates (Fig. 1c, d). This permits the cancer cells to replace the hepatocytes within the liver cell plates and allows these metastases to co-opt the sinusoidal blood vessels at the tumour–liver interface, without inducing sprouting angiogenesis. The liver cell plates can sometimes be pushed away while the cancer cells replace the hepatocytes.

### HGP categorisation

In order to investigate the hypothesis that the presence of *any* non-dHGP determines prognosis, patients were categorised as non-dHGP if any other HGP than dHGP was observed. For supplementary analyses, patients were also categorised according to the 50% cut-off value of the consensus guidelines in which case patients were categorised as dHGP, rHGP and pHGP when > 50% of the interface was scored as such. If none of the three HGP was present at > 50% of the interface, this was defined as mixed HGP and patients were excluded for further analysis, since no predominant HGP could be determined. In order to compare the angiogenic dHGP to the non-angiogenic rHGP, supplementary analyses were also performed for patients with any proportion of rHGP compared to patients with pure (100%) dHGP. To that end, patients without any rHGP in the non-dHGP group were excluded. In this way, the rare pHGP was excluded from the analyses.

### Statistical analysis method

Absolute numbers and percentages were used to present categorical data, while medians (incl. interquartile range (IQR)) were used to display continuous data. The Chi-squared test was used to evaluate differences in proportions. To compare medians between two or three groups the Mann–Whitney U or the Kruskal–Wallis test were used, respectively. Survival was estimated by means of Kaplan–Meier analysis, and the curves were computed until 60 months and compared using the logrank test. Uni- and multivariable Cox regression analysis was performed to determine if HGPs remained significantly prognostic when correcting for well-known risk factors. Results of the Cox regression analyses were expressed using hazard ratios (HR) and consequent 95% confidence intervals (CI). In order to test possible statistical interaction between neoadjuvant chemotherapy and the HGP, an interaction term was added to a multivariable Cox regression model analysing the entire study population. Other potential confounders corrected for were age, ASA score, primary tumour location, pathological T-stage, nodal status, disease-free interval, number of CRLM, diameter of the largest CRLM, carcinoembryonic antigen level, resection margin and extrahepatic disease. Uni- and multivariable binary logistic regression analysis was performed to determine whether the administration of neoadjuvant chemotherapy was associated with the HGP that was observed. Results of the logistic regression were expressed using odds ratios (OR) and corresponding 95% CI. All analyses were performed for chemo-naive and neoadjuvantly treated patients separately where applicable. Median follow-up time for survivors was estimated using the reversed Kaplan–Meier method. No imputation of missing data was applied. Schoenfeld residuals (for continuous variables) and Kaplan–Meier graphs (for categorical variables) were evaluated, in order to determine whether the proportional hazards assumption was violated. All statistical tests were two-sided and a *p* value below 0.05 was considered statistically significant. All analyses were performed using SPSS version 24.0 (SPSS Inc., Chicago, IL) and R version 3.5.1 (http://www.r-project.org).

## Results

### Patient characteristics

During the study period, 964 consecutive patients underwent laparotomy for intended first surgical treatment of CRLM. One hundred patients (10%) were excluded because no complete surgical treatment was performed. In 132 patients (15%), HGP assessment was not possible due to missing H&E tissue sections (*n* = 55), ablative therapy only (*n* = 21) or H&E tissue sections which were non-suitable for HGP determination (*n* = 56). Ultimately, the HGP could be determined in 732 patients. In 177 patients (24%), dHGP was observed and the other 555 patients (76%) all displayed to some extent a proportion of non-dHGP. A flowchart of the patient inclusion is presented in supplementary Fig. 1. Median follow-up time for the survivors was 76 months (IQR: 45–116 months), during which time 528 patients (70%) were diagnosed with disease progression and 428 patients (58%) died. Statistical interaction between neoadjuvant chemotherapy and HGP proved significant (*p* = 0.005) on multivariable analysis.

### HGP in chemo-naive patients

**Fig. 2:**
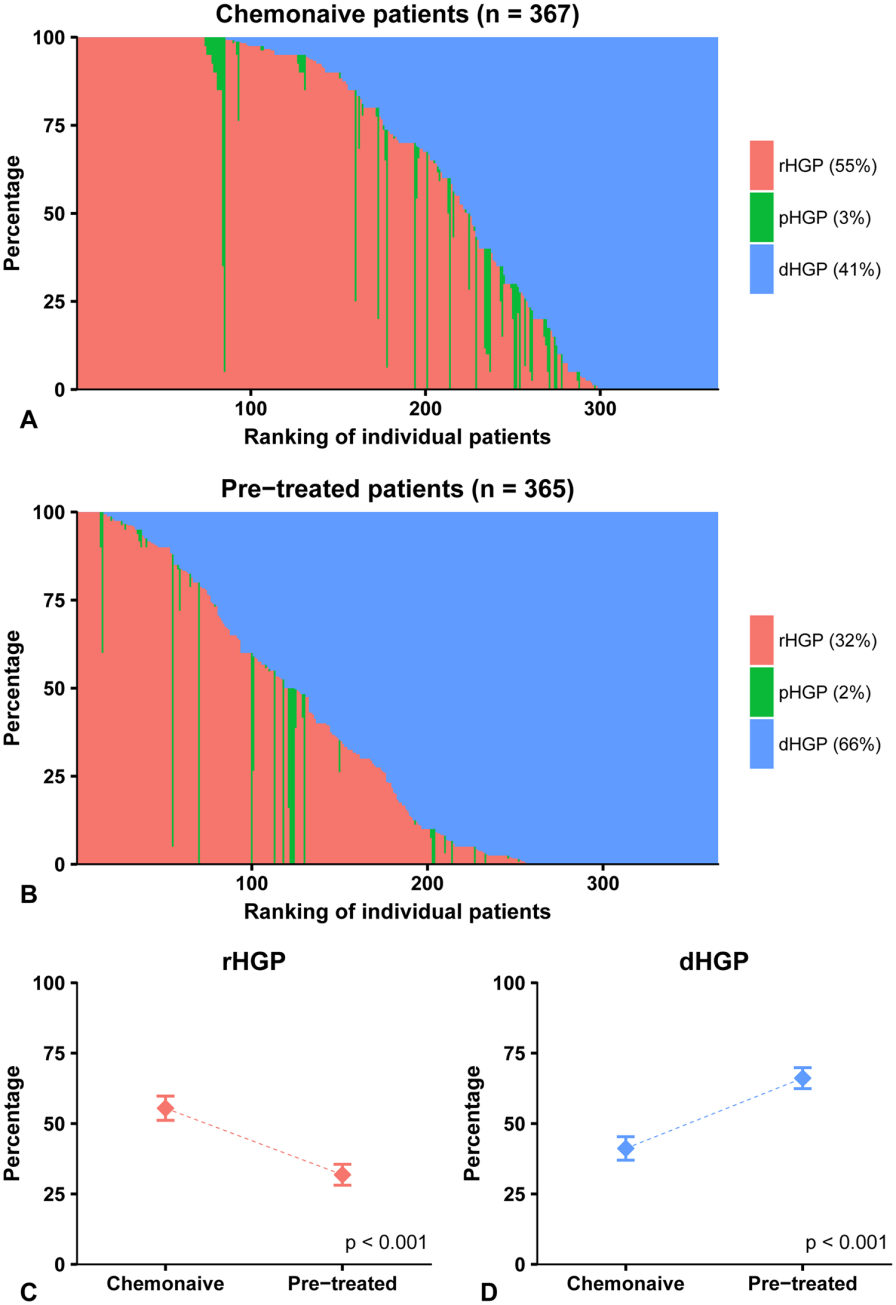
**a** Distribution of HGPs. Ranking based on percentage dHGP. **a** Distribution of HGPs in the chemo-naive cohort. **b** Distribution of HGPs in the pre-treated cohort. **c, d** Total proportion rHGP (**c**) and dHGP (**d**) in chemo-naive patients compared to pre-treated patients (percentages do not always add up to 100% due to rounding)

Of the 732 patients assessed, 367 (50%) did not receive neoadjuvant chemotherapy. In this subgroup of patients, 68 (19%) displayed dHGP only while *n* = 214 (58%) displayed dHGP in combination with non-dHGP, and *n* = 85 (23%) displayed no dHGP. In total, 299 patients (81%) displayed some proportion of non-dHGP (Fig. 2a). Baseline characteristics compared for the presence of any non-dHGP are displayed in supplementary table 1.

**Fig. 3:**
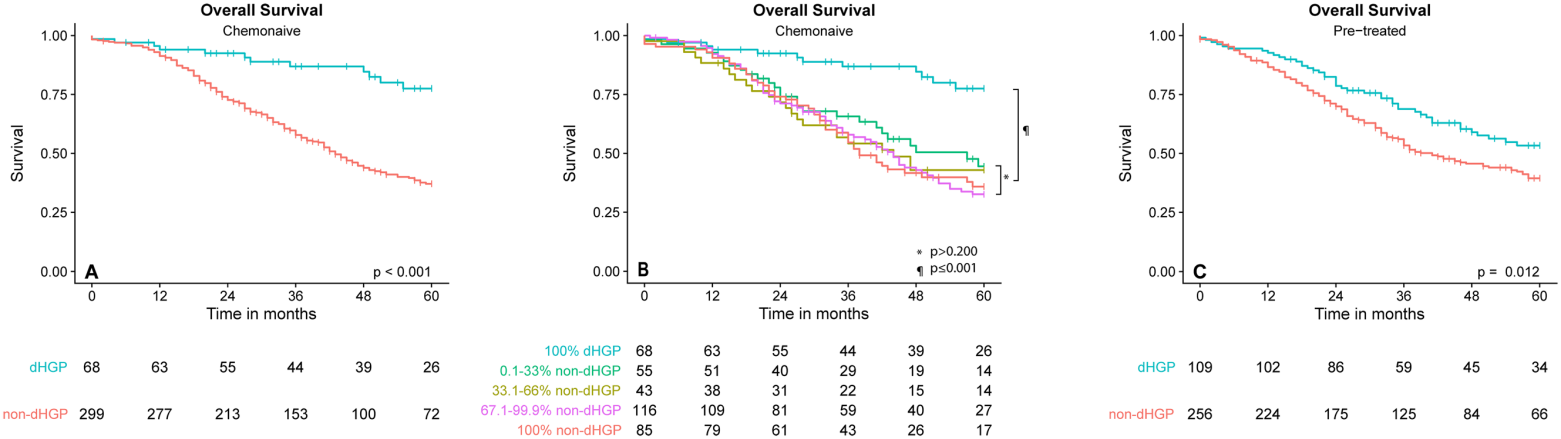
**a** OS chemo-naive patients; **b** Cut-off analysis with OS for percentage dHGP amongst chemo-naive patients; **c** pre-treated patients

**Fig. 4:**
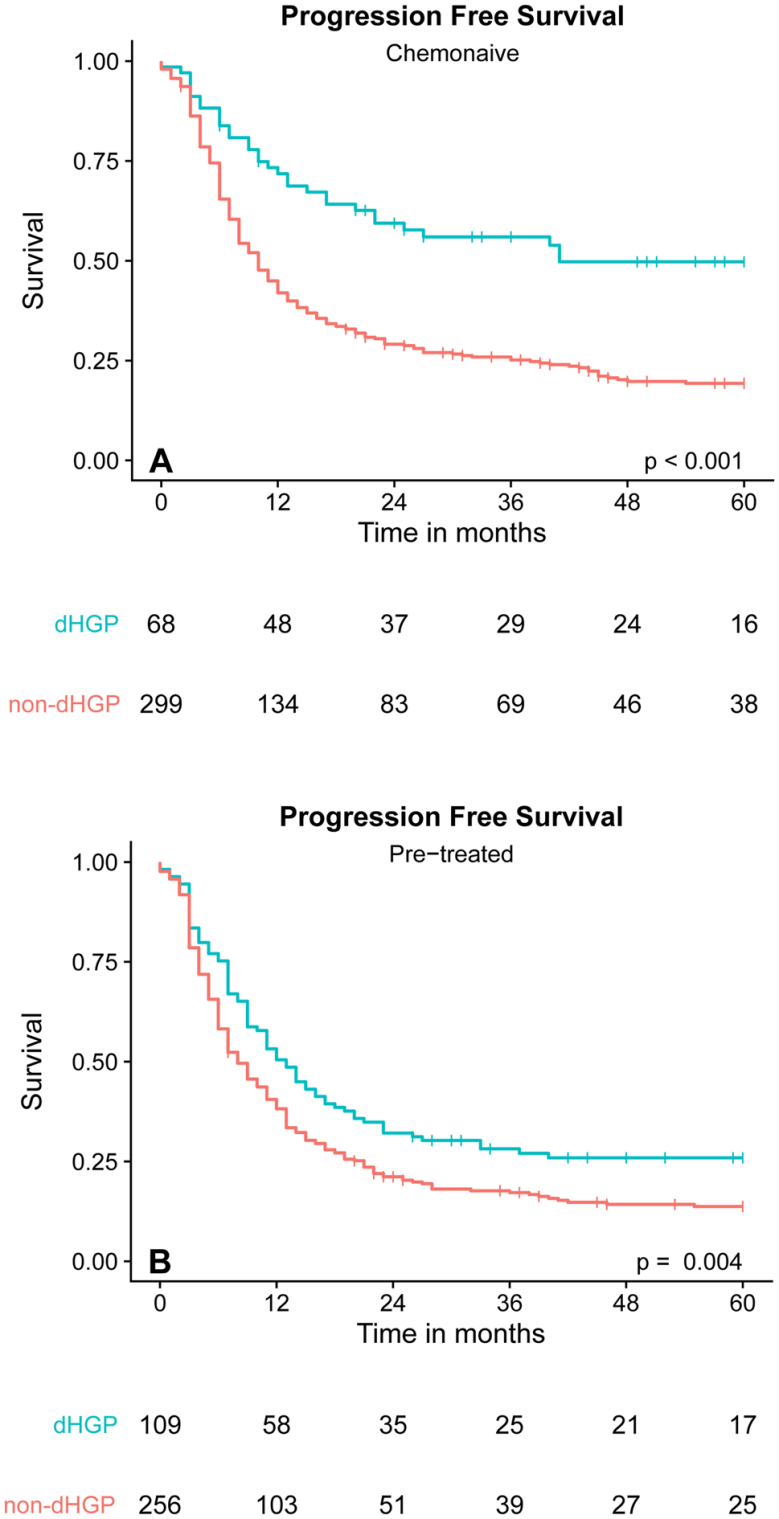
**a** PFS of chemo-naive patients. **b** PFS of pre-treated patients

Patients with dHGP had a 5-year OS rate of 78% compared to 37% of patients with any non-dHGP (Fig. 3a). After correction for potential confounders, dHGP remained significantly associated with improved OS (adjusted HR 0.39, *p* < 0.001) compared to non-dHGP (Table 1). Similar results were obtained for PFS. The 5-year PFS rate of patients with dHGP was 50% compared to 19% of patients with any non-dHGP. On multivariable analysis dHGP also remained significantly associated with improved PFS (adjusted HR 0.54, *p* = 0.001) (Table 1; Fig. 4a).

**Table 1:** Cox regression analysis for OS and PFS of chemo-naive patients

Overall survival	Univariable		Multivariable	
Variable	Hazard ratio [95% CI]	*p* Value	Hazard ratio [95% CI]	*p* Value
Age at resection CRLM (cont.)	1.011 [0.997–1.025]	0.126	1.016 [1.002–1.032]	0.030
ASA > II	1.018 [0.648–1.600]	0.939	0.985 [0.614–1.579]	0.949
Right-sided primary	1.477 [1.053–2.072]	0.024	1.539 [1.074–2.207]	0.019
pT3-4	1.191 [0.852–1.666]	0.306	0.902 [0.626–1.300]	0.579
Node-positive primary	1.459 [1.102–1.933]	0.008	1.570 [1.140–2.164]	0.006
Disease-free interval (cont.)	0.997 [0.991–1.004]	0.454	0.990 [0.983–0.998]	0.011
Number of CRLM (cont.)	1.145 [1.031–1.273]	0.012	1.095 [0.969–1.237]	0.144
Diameter largest CRLM (cont.)	1.099 [1.041–1.162]	< 0.001	1.102 [1.026–1.185]	0.008
Preoperative CEA level (cont.)	1.001 [1.000–1.002]	0.003	1.001 [1.000–1.002]	0.063
R1 resection CRLM	1.321 [0.892–1.956]	0.165	1.116 [0.738–1.685]	0.603
Extra hepatic disease	1.495 [0.896–2.496]	0.124	1.688 [0.930–3.066]	0.085
dHGP	0.314 [0.191–0.515]	< 0.001	0.394 [0.233–0.667]	< 0.001

Progression-free survival	Univariable		Multivariable	
Variable	Hazard ratio [95% CI]	*p* Value	Hazard ratio [95% CI]	*p* Value
Age at resection CRLM (cont.)	0.998 [0.987–1.010]	0.769	1.005 [0.993–1.018]	0.387
ASA > II	0.836 [0.554–1.262]	0.394	0.852 [0.555–1.306]	0.462
Right-sided primary	1.179 [0.868–1.602]	0.291	1.232 [0.893–1.698]	0.204
pT3-4	1.175 [0.878–1.573]	0.279	0.873 [0.634–1.203]	0.407
Node-positive primary	1.566 [1.224–2.002]	< 0.001	1.558 [1.184–2.049]	0.002
Disease-free interval (cont.)	0.993 [0.986-1.000]	0.039	0.989 [0.981–0.996]	0.003
Number of CRLM (cont.)	1.215 [1.102–1.340]	< 0.001	1.150 [1.029–1.285]	0.013
Diameter largest CRLM (cont.)	1.026 [0.972–1.083]	0.345	1.036 [0.970–1.107]	0.287
Preoperative CEA level (cont.)	1.001 [1.000-1.002]	0.041	1.001 [1.000-1.002]	0.167
R1 resection CRLM	1.620 [1.149–2.285]	0.006	1.376 [0.956–1.982]	0.086
Extra hepatic disease	1.199 [0.760–1.892]	0.434	1.596 [0.953–2.672]	0.076
dHGP	0.452 [0.317–0.645]	< 0.001	0.536 [0.366–0.786]	0.001

*ASA* American Society of Anaesthesiologists, *CEA* carcinoembryonic antigen, *cont*. continuous, *CRLM* colorectal liver metastases, *dHGP* desmoplastic histopathological growth pattern, *R1* irradical resection margin

When the OS for different percentages of non-dHGP was evaluated (Fig. 3b), there were no differences in OS between patients who displayed any non-dHGP, regardless of the percentage of non-dHGP (all *p* values > 0.2). Kaplan–Meier analysis showed that all patients with any non-dHGP had significantly impaired survival compared to patients who had (100%) dHGP (all *p* values ≤ 0.001). This finding was confirmed on multivariable analysis (Table 2).

**Table 2:** Overall survival cox regression cut-off analysis in the chemo-naive cohort

Overall survival	Univariable		Multivariable	
Variables	Hazard ratio [95% CI]	*p* Value	Hazard ratio [95% CI]	*p* Value
Age at resection CRLM (cont.)	1.011 [0.997–1.025]	0.126	1.017 [1.002–1.032]	0.031
ASA > II	1.018 [0.648–1.600]	0.939	0.968 [0.600–1.564]	0.896
Right-sided primary	1.477 [1.053–2.072]	0.024	1.563 [1.088–2.247]	0.016
pT3-4	1.191 [0.852–1.666]	0.306	0.890 [0.617–1.285]	0.535
Node-positive primary	1.459 [1.102–1.933]	0.008	1.583 [1.142–2.194]	0.006
Disease-free interval (cont.)	0.997 [0.991–1.004]	0.454	0.990 [0.982–0.998]	0.010
Number of CRLM (cont.)	1.145 [1.031–1.273]	0.012	1.104 [0.974–1.252]	0.122
Diameter largest CRLM (cont.)	1.099 [1.041–1.162]	< 0.001	1.105 [1.026–1.189]	0.008
Preoperative CEA level (cont.)	1.001 [1.000–1.002]	0.003	1.001 [1.000–1.002]	0.103
R1 resection CRLM	1.321 [0.892–1.956]	0.165	1.103 [0.727–1.671]	0.645
Extra hepatic disease	1.495 [0.896–2.496]	0.124	1.627 [0.886–2.987]	0.116
100% dHGP	Ref		Ref	
0.1–33% non-dHGP	2.851 [1.582–5.137]	< 0.001	2.350 [1.248–4.425]	0.008
33.1–67% non-dHGP	2.840 [1.547–5.215]	< 0.001	2.458 [1.303–4.639]	0.005
67.1–99.9% non-dHGP	3.255 [1.924–5.505]	< 0.001	2.443 [1.393–4.284]	0.002
100% non-dHGP	3.535 [2.055–6.084]	< 0.001	2.858 [1.605–5.088]	< 0.001

### HGP in neoadjuvantly treated patients

In total, 365 patients (50%) received chemotherapy within 6 months prior to liver resection. The distribution of HGPs amongst neoadjuvantly treated patients is displayed in Fig. 2b. Baseline characteristics of neoadjuvantly treated patients compared for the presence of any non-dHGP are displayed in supplementary table 2. Patients who were treated neoadjuvantly with chemotherapy had a more severe disease burden (Supplementary table 3). The chemotherapeutic regimen was oxaliplatin-based in 309 patients (85%) and irinotecan based in 34 (9%). Fifteen patients received a 5-Fluorouracil derivative only (4%). Six patients (2%) received a combination of oxaliplatin and irinotecan and in one patient the type of chemotherapy was unknown. In 119 patients (33%), bevacizumab was added to the chemotherapy regimen.

Of the neoadjuvantly treated patients, 109 (30%) had dHGP and 256 (70%) displayed a proportion of non-dHGP (Fig. 2b). dHGP was observed in a greater number of samples from neoadjuvantly treated than chemo-naive patients (30% vs. 19%, *p* < 0.001). The total proportion of any dHGP in neoadjuvantly treated patients was 66%, while this was 41% in chemo-naive patients. A similar difference was observed for the total proportion of any rHGP (both *p* < 0.001, Fig. 2c, d). The association between neoadjuvant chemotherapy and the presence of dHGP remained significant (adjusted OR 2.71, *p* < 0.001) after correction for several clinicopathological characteristics (Supplementary table 4).

The addition of bevacizumab to the chemotherapeutic regimen was not associated with a significant increase of the proportion of dHGP (35% vs. 27%, *p* = 0.120). A subsequently performed multivariable logistic regression model failed to demonstrate a significant association between dHGP and the administration of bevacizumab (adjusted OR 1.60, *p* = 0.077) (Supplementary table 5).

Five-year OS in neoadjuvantly treated patients with dHGP was 53%, while a 5-year OS of 40% was seen in patients with non-dHGP (Fig. 3c, *p* = 0.012). When correcting for confounders, no significant association of dHGP was observed for OS (adjusted HR 0.98, *p* = 0.623) (Table 3). Again, similar results were obtained for the PFS. Neoadjuvantly treated patients with dHGP had a 5-year PFS rate of 26% compared to 14% in patients with non-dHGP (Fig. 4b, *p* = 0.004). On multivariable analysis, only a trend towards a significant association of dHGP with PFS was seen (adjusted HR 0.76, *p* = 0.065) (Table 3).

**Table 3:** Cox regression analysis for OS and PFS of pre-treated patients

Overall survival	Univariable		Multivariable	
Variable	Hazard ratio [95% CI]	*p* Value	Hazard ratio [95% CI]	*p* Value
Age at resection CRLM (cont.)	1.021 [1.007–1.036]	0.003	1.034 [1.016–1.051]	< 0.001
ASA > II	1.082 [0.675–1.733]	0.744	1.197 [0.728–1.968]	0.479
Right-sided primary	0.877 [0.590–1.304]	0.517	0.954 [0.624–1.459]	0.829
pT3-4	1.476 [0.988–2.204]	0.057	1.402 [0.900–2.183]	0.135
Node-positive primary	1.419 [1.050–1.918]	0.023	1.383 [0.994–1.923]	0.054
Disease-free interval (cont.)	0.996 [0.985–1.008]	0.541	0.995 [0.982–1.008]	0.452
Number of CRLM (cont.)	1.023 [0.976–1.072]	0.340	1.051 [0.995–1.111]	0.076
Diameter largest CRLM (cont.)	0.997 [0.952–1.045]	0.905	1.026 [0.969–1.086]	0.381
Preoperative CEA level (cont.)	1.000 [1.000–1.000]	0.955	1.000 [1.000–1.000]	0.574
R1 resection CRLM	1.374 [0.989–1.908]	0.058	1.273 [0.867–1.869]	0.218
Extra hepatic disease	1.705 [1.222–2.380]	0.002	1.761 [1.196–2.592]	0.004
dHGP	0.661 [0.484–0.902]	0.009	0.915 [0.643–1.302]	0.623

Progression-free survival	Univariable		Multivariable	
Variable	Hazard ratio [95% CI]	*p* Value	Hazard ratio [95% CI]	*p* Value
Age at resection CRLM (cont.)	1.008 [0.996–1.019]	0.188	1.011 [0.998–1.025]	0.106
ASA > II	1.086 [0.731–1.614]	0.682	1.045 [0.682–1.600]	0.840
Right-sided primary	0.936 [0.684–1.282]	0.681	1.053 [0.752–1.474]	0.764
pT3-4	1.420 [1.021–1.974]	0.037	1.440 [1.005–2.065]	0.047
Node-positive primary	1.328 [1.032–1.710]	0.028	1.143 [0.869–1.501]	0.339
Disease-free interval (cont.)	0.994 [0.985–1.004]	0.234	0.996 [0.986–1.007]	0.462
Number of CRLM (cont.)	1.026 [0.989–1.063]	0.174	1.036 [0.992–1.081]	0.109
Diameter largest CRLM (cont.)	0.993 [0.954–1.034]	0.728	1.000 [0.955–1.048]	0.986
Preoperative CEA level (cont.)	1.000 [1.000–1.000]	0.462	1.000 [1.000–1.000]	0.443
R1 resection CRLM	1.464 [1.101–1.948]	0.009	1.449 [1.043–2.015]	0.027
Extra hepatic disease	1.777 [1.321–2.390]	< 0.001	1.912 [1.367–2.674]	< 0.001
dHGP	0.671 [0.519–0.867]	0.002	0.762 [0.570–1.017]	0.065

*ASA* American Society of Anaesthesiologists, *CEA* carcinoembryonic antigen, *cont*. continuous, *CRLM* colorectal liver metastases, *dHGP* desmoplastic histopathological growth pattern, *R1* irradical resection margin

Additional Kaplan–Meier analyses showed no overall survival difference when adding bevacizumab to the chemotherapeutic regimen in the total group (*p* = 0.754), in the non-dHGP (*p* = 0.854) or in the dHGP subgroup (*p* = 0.411). Similar results were found for PFS in the total group (*p* = 0.806), the non-dHGP (*p* = 0.829) or the dHGP subgroup (*p* = 0.806). Subsequent multivariable analysis in the total neoadjuvantly treated group with bevacizumab entered as potential confounder showed no significant association of bevacizumab with OS (adjusted HR 1.06, *p* = 0.702; supplementary table 6.) or PFS (adjusted HR 1.09, *p* = 0.540; supplementary table 7).

### Consensus cut-off

Supplementary analyses performed using the consensus guidelines > 50% predominant HGP cut-off confirmed results: superior survival for dHGP, higher proportion of dHGP after neoadjuvant chemotherapy and loss of prognostic impact of dHGP in neoadjuvantly treated patients. These data are presented in supplementary tables 8–12 and supplementary Fig. 2a, b.

### dHGP versus any rHGP

In order to make a direct comparison of angiogenic dHGP versus non-angiogenic rHGP growth, we have performed separate, supplementary analyses which excluded the few cases with angiogenic pHGP. Patients with any proportion of rHGP were compared to patients with pure (100%) dHGP, excluding patients without any rHGP from the non-dHGP group. In total, 26 patients, of which 13 were chemo-naive, without rHGP were observed in the non-dHGP group and excluded for these analyses. Again, all analyses had similar results: superior OS (HR 0.40, *p* < 0.001) and PFS (HR 0.55, *p* = 0.002) for chemo-naive patients with dHGP and a reduced prognostic impact of HGPs after neoadjuvant chemotherapy (OS—HR: 0.88, *p* = 0.505; PFS—HR: 0.73, *p* = 0.032).

### HGP comparison of multiple hepatectomies

**Fig. 5:**
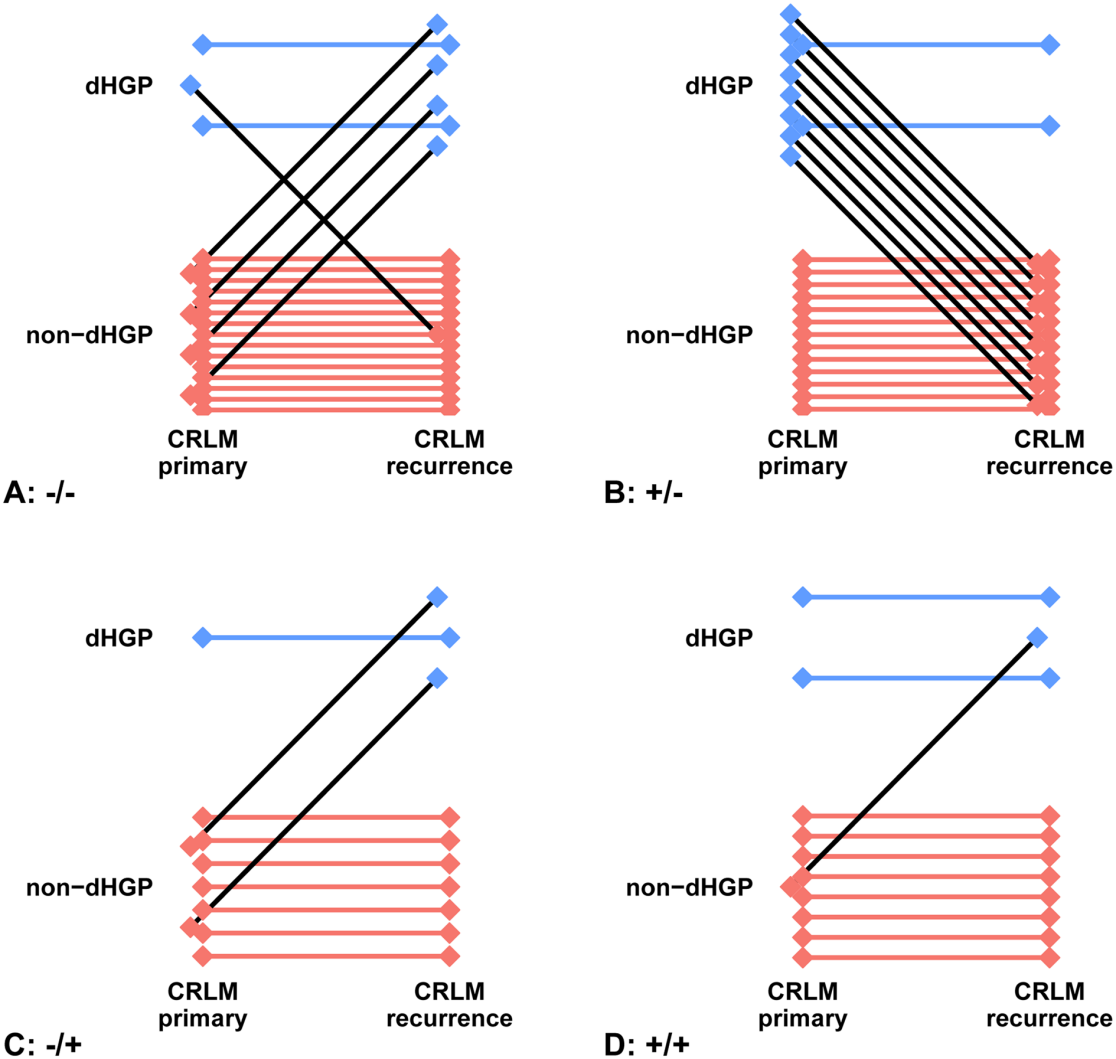
**a**–**d** Graphical display of the changes in HGPs between first and second surgery for CRLM found per group: **a** −/−; **b** +/−; **c** −/+; **d** +/+

Amongst the included patients, the HGP of recurrent CRLM could be determined in 66 patients. A similar proportional distribution of HGPs was observed in these patients. After surgery for recurrent CRLM without neoadjuvant chemotherapy, dHGP tumours were found in 18% (8/45) of patients, compared to 29% (6/21) in patients who did receive chemotherapy (*p* = 0.318). Four groups (−/−, +/−, −/+, +/+), as described in the methods, were created. Figure 5a–d graphically displays the changes in HGPs found per group. The difference in proportion HGPs between the first and second surgery was significant in the +/− group (Fig. 5b, *p* = 0.007). The other changes in proportions of HGP between the first and second surgery were not significant (all *p* values > 0.250).

## Discussion

The current study demonstrates that HGPs have significant prognostic potential for colorectal cancer patients who undergo first resection of CRLM. Our results indicate that in chemo-naive patients the presence of a pure dHGP predicts improved survival with a hazard ratio unmatched by any clinicopathological or biological correlate to date [12, 13]. This is the first study to show that the presence of *any* non-dHGP is sufficient to indicate impaired prognosis. Interestingly, chemotherapy is associated with an increased incidence of CRLM displaying dHGP in the current patient cohort and the prognostic impact of dHGP is reduced in these patients.

Stratifying patient groups for preoperative treatment status showed that the proportion and prognostic impact of HGPs differs significantly between chemo-naive and neoadjuvantly treated patients. Previous studies examined relatively small and heterogeneous patient groups which hampered adequate multivariable analysis, whereas the large number of events in the current study ensured that proper correction for confounders could be performed [4, 7–10]. In addition, preceding studies did not perform cut-off analyses for different proportions of HGPs. The currently performed cut-off analysis showed that an increasing proportion of non-dHGP was not associated with a decrease in prognosis. Therefore, the presence of any non-dHGP, rather than the actual proportion of the tumour–liver interface occupied by non-dHGP, dictates worse survival compared to patients with 100% dHGP. This suggests that an arbitrary cut-off should not be applied to define the non-dHGP growth pattern. This information can be integrated in future consensus guidelines for scoring the HGPs of CRLM.

Neoadjuvant chemotherapy (with or without bevacizumab) has been associated with tumoural fibrosis and necrosis in CRLM [14, 15]. Treatment with bevacizumab has been associated with alterations in the extracellular matrix (ECM) of CRLM [16] and the ECM has been argued to influence the hallmarks of cancer [17]. Given these associations, one could hypothesise that treating CRLM with chemotherapy with or without bevacizumab could induce alterations in the HGP. In the current study, it has been possible to determine the prevalence of different HGP types in CRLM relative to chemotherapy status and with the addition of bevacizumab. We observed a higher proportion of 100%dHGP in neoadjuvantly treated patients, but the prognostic impact of this growth pattern was relatively reduced in this patient category. Similar results were found within the subgroup in whom bevacizumab was added to the chemotherapy regimen, but this was not significantly different compared to the group that was treated neoadjuvantly without bevacizumab. Moreover, the previously reported survival benefit of the addition of bevacizumab to chemotherapy in 51 patients with dHGP [4] could not be demonstrated in the current study. At our institution, evident progressive disease during chemotherapy is a contra-indication for surgical treatment of CRLM. As poor pathological and radiological response is associated with rHGP [4], it is possible that progressive patients have CRLM displaying non-dHGP. This could have resulted in a higher relative proportion of dHGP in the neoadjuvantly treated patient cohort. Unfortunately, data on the percentage of patients that were not operated upon because of disease progression are unavailable in our series. In randomised setting, approximately 7% of patients with resectable CRLM display progressive disease during chemotherapy [18]. In addition, considerable differences in clinical risk were seen when comparing chemo-naive patients with neoadjuvantly treated patients in this non-randomised cohort. An alternative explanation for both the larger proportion of dHGP and the reduced prognostic impact of HGPs in the neoadjuvantly treated cohort is that a biological response to chemotherapy is a histological conversion to dHGP, the relevance of which we have yet to determine. Of patients considered chemo-naive for their recurrent CRLM, 18% (8/45) had recurrent CRLM displaying dHGP compared to 29% (6/21) in patients treated neoadjuvantly for their recurrent CRLM. This difference in proportional distribution of recurrent HGPs was not significant. Nevertheless, it was similar to the proportional distribution of HGPs observed after first hepatectomy in which the difference was significant. When taking neoadjuvant treatment status of both resections into account, in the +/− group 35% (8/23) changed from dHGP (first surgery) to non-dHGP (second surgery), while this change was only seen in 5% (1/22) of the −/− group. These data could support the hypothesis of potential conversion of the HGP as a consequence of chemotherapy. An alternative explanation for this observation could be that patients who at first have dHGP CRLM, but develop non-dHGP CRLM at recurrence as the disease might acquire a more aggressive tumour biology. In addition, Frentzas et al. also found a relatively large proportion of rHGP in recurrent CRLM, albeit after combination therapy of chemotherapy and bevacizumab for the recurrent CRLM [4]. The value of these data remains limited, because of its retrospective nature, selected population and low patient numbers. Further study of the HGPs in chemo-naive versus neoadjuvantly treated CRLM is required to investigate this concept and, more specifically, data from randomised studies will be needed to further evaluate this hypothesis.

The biological mechanisms that underlie the association of non-dHGP with impaired survival remain largely unknown. The non-dHGP cohort in this study consists almost exclusively of patients with liver metastases that display the vessel co-opting, non-angiogenic rHGP. An important difference between rHGP and dHGP is indeed the mechanism of vascularisation. The desmoplastic growth pattern of liver metastases has an elevated fraction of proliferating endothelial cells and blood vessels are organised in vascular hot spots [3, 19], both clear features of angiogenesis. The vascular architecture of the metastasis does not resemble the vascular architecture of the adjacent liver tissue. These findings also apply to the pushing growth pattern. In the replacement growth pattern, on the contrary, a low endothelial cell proliferation fraction and a lack of vascular hot spots are observed [3, 19]. The tumour tissue mimics the liver tissue by growing along and using the sinusoidal blood vessels. The preservation of the normal tissue architecture is indicative of non-angiogenic tumour growth. The co-opted capillary bed from normal liver is highly efficient and liver metastases with a rHGP display minimal hypoxia and vascular leakage as opposed to the desmoplastic liver metastases with their vasculature created in an angiogenic environment in which tortuous, disrupted, leaking and dysfunctional blood vessels result in hypoxia [3]. The association between growth patterns and the means of tumour vascularisation (by angiogenesis or by vessel co-option) is not limited to tumour growth in the liver, but has also been described in, for example, the lungs, the lymph nodes and the skin [20]. The motile and invasive cancer cells present in replacement metastases enables the incorporation of normal surrounding tissue stroma and creates the typical irregular tumour border. Up-regulation of signalling pathways of cell motility has been described in pre-clinical models of CRC liver metastases and primary liver cancer [4, 21]. Similarly, molecular signatures of cancer cell motility and invasion have been identified in angiotropism, a process of peri-vascular growth that closely resembles vascular co-option during replacement growth [22, 23]. Co-localisation of cancer cells and endothelial cells during vascular co-option also results in angiocrine signalling. Soluble ligands of the notch-pathway produced by endothelial cells induce stemness in adjacent cancer cells which is associated with both cancer cell motility and with resistance to chemotherapy [24]. Again, similar observations have been reported for angiotropic tumours [23]. Beyond the intrinsic changes in the tumour and stroma observed in replacement metastases, an effective immune response in patients with dHGP also might contribute to the difference in survival outcomes between these two HGPs [5, 25]. Brunner et al. demonstrated that capsule formation in dHGP strongly correlates with high levels of peri-tumour infiltration of CD4+, CD45RO+ and CD8+ cells [5]. Taken together, these findings corroborate the less favourable prognosis of patients with liver metastases that have the ability to perform non-desmoplastic growth.

For a more direct comparison of angiogenic dHGP and non-angiogenic rHGP growth, we have excluded the few cases with angiogenic pHGP in separate analyses. Non-angiogenic replacement HGP has been associated with aggressive tumour growth in which normal sinusoidal liver capillaries are co-opted by the metastasis. The pHGP can be difficult to distinguish from the rHGP when during replacement growth the liver cell plates are also pushed aside. This HGP assessment problem has been extensively addressed in the international consensus for scoring the histopathological growth patterns of liver metastases [2]. This, however, is an additional reason to selectively study the impact on survival of pure (100%) dHGP. It will be necessary to assemble a large cohort of patients with pHGP to accurately study the impact of this growth pattern on outcome.

The results of the current study should be interpreted in the light of its limitations. The HGP data were collected retrospectively, in 55 potentially eligible patients tissue sections were missing and there were 56 patients with unsuitable H&E tissue sections. It was also not possible to examine CRLM from patients with progressive disease during chemotherapy as this as a contra-indication for surgical treatment at our institution. This study was also limited by the unavailability of RAS and BRAF mutational status. Both mutations have been suggested as prognostic biomarkers for survival after liver resection for CRLM [13, 26, 27] In addition, Brudvik et al. proposed an enhanced clinical risk score, including the RAS mutational status. The authors demonstrated improved performance of the prognostic model [28]. In an attempt to overcome this shortcoming, the current study was corrected for right-sidedness of the primary tumour, which is associated with KRAS [29, 30] and BRAF [29–31] mutational status.

In conclusion, the current study demonstrates in the largest patient cohort to date with multivariable analyses that HGPs, distinguishing angiogenic from non-angiogenic growth, have considerable prognostic impact in patients who are treated surgically for CRLM. The presence of *any* non-desmoplastic, non-angiogenic HGP displaying vessel co-opting growth, rather than the actual proportion of non-dHGP, determines prognosis suggesting that future studies and guidelines should focus upon this distinction.

## Electronic supplementary material

Below is the link to the electronic supplementary material.

Supplementary table 1. Baseline characteristics compared for the presence of any non-dHGP (DOCX 17 KB)

Supplementary table 2. Baseline characteristics pre-treated patients dHGP vs non-dHGP (DOCX 17 KB)

Supplementary table 3. Baseline characteristics chemo-naive versus pre-treated patients (DOCX 19 KB)

Supplementary table 4. Uni- and multivariable logistic regression analysis for association with dHGP (DOCX 14 KB)

Supplementary table 5. (Rebuttal table 2.) Uni- and multivariable logistic regression analysis for association with dHGP in the neoadjuvantly treated group (DOCX 14 KB)

Supplementary table 6. Overall Survival Cox regression analysis all neoadjuvantly treated patients +/- Bevacizumab (DOCX 14 KB)

Supplementary table 7. Progression-Free Survival Cox regression all neoadjuvantly treated patients +/- Bevacizumab (DOCX 14 KB)

Supplementary table 8. Baseline characteristics chemo-naive patients 50% cut-off (DOCX 18 KB)

Supplementary table 9. Uni- and multivariable Cox regression analysis OS of chemo-naive patients >50% cut-off (DOCX 15 KB)

Supplementary table 10. Uni- and multivariable logistic regression analysis for association with dHGP >50% cut-off (DOCX 14 KB)

Supplementary table 11. Baseline characteristics pre-treated patients >50% cut-off (DOCX 18 KB)

Supplementary table 12. Uni- and multivariable Cox regression analysis for OS of pre-treated patients >50% cut-off (DOCX 15 KB)

Supplementary figure 1. A flowchart of the patient inclusion (TIF 15495 KB)

Supplementary figure 2A-B. OS using the >50% cut-off. 2A: OS chemo-naive patients. 2B: OS pre-treated patients (TIF 1177 KB)
