# Supplementary material for: Angiogenic desmoplastic histopathological growth pattern as a prognostic marker of good outcome in patients with colorectal liver metastases
Source: Angiogenesis. 2019 Jan 12;22(2):355–68. doi: 10.1007/s10456-019-09661-5 (PMC6475515; doi:10.1007/s10456-019-09661-5)
Supplement: Supplementary file 1 — Supplementary table 1. Baseline characteristics compared for the presence of any non-dHGP (DOCX 17 KB) [file 10456_2019_9661_MOESM1_ESM.docx]

| **Supplementary table 1. Baseline characteristics chemo-naive patients dHGP vs non-dHGP** | | | | |
| --- | --- | --- | --- | --- |
|  |  | **dHGP** | **non-dHGP** | **p-value** |
|  |  | **N=68 (19%)** | **N=299 (81%)** |  |
| **General characteristics** |  |  |  |  |
| Age at resection (median [IQR]) |  | 68.0 [59.0, 75.2] | 66.0 [59.0, 73.0] | 0.180 |
| Gender (%) | Female | 25 (37) | 109 (36) | 0.962 |
|  | Male | 43 (63) | 190 (64) |  |
| ASA classification(%) | ASA Class I-II | 57 (85) | 265 (91) | 0.142 |
|  | ASA Class >II | 10 (15) | 26 (9) |  |
|  | *Missing (N=9)* |  |  |  |
| **Primary tumour characteristics** |  |  |  |  |
| Location (%) | Right-sided | 11 (16) | 51 (17) | 0.468 |
|  | Left-sided | 32 (47) | 115 (38) |  |
|  | Rectum | 22 (32) | 124 (41) |  |
|  | Double tumour | 3 (4) | 9 (3) |  |
| Pathological T-stage (%) | pT 0-2 | 19 (28) | 61 (21) | 0.188 |
|  | pT 3-4 | 49 (72) | 235 (79) |  |
|  | *Missing (N=3)* |  |  |  |
| Pathological N-stage (%) | N0 | 35 (52) | 118 (40) | 0.070 |
|  | N+ | 32 (48) | 176 (60) |  |
|  | *Missing (N=6)* |  |  |  |
| Adjuvant chemotherapy (%) | No | 59 (87) | 231 (77) | 0.082 |
|  | Yes | 9 (13) | 68 (23) |  |
| **CRLM characteristics** |  |  |  |  |
| Synchronous CRLM (%) | Metachronous | 43 (63) | 212 (71) | 0.215 |
|  | Synchronous | 25 (37) | 87 (29) |  |
| DFI (median [IQR]) |  | 10.0 [0.0, 20.0] | 13.0 [0.5, 25.5] | 0.154 |
| Number of CRLM (median [IQR]) |  | 1.0 [1.0, 2.0] | 1.0 [1.0, 2.0] | 0.195 |
| Largest diameter CRLM (median [IQR]) | *Missing (N=1)* | 2.0 [1.5, 3.6] | 3.2 [2.2, 4.1] | <0.001* |
| Preoperative CEA (median [IQR]) | *Missing (N=14)* | 6.0 [3.0, 12.0] | 13.6 [4.7, 44.2] | <0.001* |
| Bilobar (%) | Unilobar | 54 (79) | 232 (78) | 0.744 |
|  | Bilobar | 14 (21) | 67 (22) |  |
| Extrahepatic disease (%) | No | 64 (94) | 277 (93) | 0.669 |
|  | Yes | 4 (6) | 22 (7) |  |
| Resection margin (%) | R0 | 65 (96) | 259 (87) | 0.048* |
|  | R1 | 3 (4) | 38 (13) |  |
|  | *Missing (N=2)* |  |  |  |
| CRS (%) | Low (0-2) | 53 (80) | 230 (78) | 0.746 |
|  | High (3-5) | 13 (20) | 63 (22) |  |
|  | *Incomplete (N=8)* |  |  |  |
| Major resection (≥3 complete segments) (%) | No major resection | 59 (87) | 226 (76) | 0.046* |
|  | Major resection | 9 (13) | 73 (24) |  |
| Major complications (i.e. Clavien-Dindo ≥3) | No | 61 (90) | 277 (93) | 0.363 |
|  | Yes | 7 (10) | 21 (7) |  |
|  | *Missing (N=1)* |  |  |  |
| Postoperative death (%) | No | 67 (99) | 293 (98) | 0.770 |
|  | Yes | 1 (1) | 6 (2) |  |
