# Supplementary material for: Angiogenic desmoplastic histopathological growth pattern as a prognostic marker of good outcome in patients with colorectal liver metastases
Source: Angiogenesis. 2019 Jan 12;22(2):355–68. doi: 10.1007/s10456-019-09661-5 (PMC6475515; doi:10.1007/s10456-019-09661-5)
Supplement: Supplementary file 2 — Supplementary table 2. Baseline characteristics pre-treated patients dHGP vs non-dHGP (DOCX 17 KB) [file 10456_2019_9661_MOESM2_ESM.docx]

| **Supplementary table 2. Baseline characteristics pre-treated patients dHGP vs non-dHGP** | | | | |
| --- | --- | --- | --- | --- |
|  |  | **dHGP** | **non-dHGP** | **p-value** |
|  |  | **N=109 (30%)** | **N=256 (70%)** |  |
| **General characteristics** |  |  |  |  |
| Age at resection (median [IQR]) |  | 63.0 [55.0, 70.0] | 63.0 [56.8, 68.2] | 0.858 |
| Gender (%) | Female | 40 (37) | 89 (35) | 0.724 |
|  | Male | 69 (63) | 167 (65) |  |
| ASA classification(%) | ASA Class I-II | 100 (92) | 234 (92) | 0.995 |
|  | ASA Class >II | 9 (8) | 21 (8) |  |
|  | *Missing (N=1)* |  |  |  |
| **Primary tumour characteristics** |  |  |  |  |
| Location (%) | Right-sided | 20 (18) | 38 (15) | 0.657 |
|  | Left-sided | 45 (41) | 115 (45) |  |
|  | Rectum | 42 (39) | 101 (39) |  |
|  | Double tumour | 2 (2) | 2 (1) |  |
| Pathological T-stage (%) | pT 0-2 | 20 (19) | 36 (16) | 0.483 |
|  | pT 3-4 | 86 (81) | 192 (84) |  |
|  | *Missing (N=31)* |  |  |  |
| Pathological N-stage (%) | N0 | 44 (42) | 77 (34) | 0.152 |
|  | N+ | 61 (58) | 151 (66) |  |
|  | *Missing (N=32)* |  |  |  |
| Adjuvant chemotherapy (%) | No | 105 (96) | 228 (90) | 0.056 |
|  | Yes | 4 (4) | 24 (10) |  |
|  | *Missing (N=4)* |  |  |  |
| **CRLM characteristics** |  |  |  |  |
| Synchronous CRLM (%) | Metachronous | 22 (20) | 60 (23) | 0.495 |
|  | Synchronous | 87 (80) | 196 (77) |  |
| DFI (median [IQR]) |  | 0.0 [0.0, 2.0] | 0.0 [0.0, 2.2] | 0.822 |
| Number of CRLM (median [IQR]) |  | 3.0 [1.0, 4.0] | 3.0 [2.0, 5.0] | 0.018 |
| Largest diameter CRLM (median [IQR]) | *Missing (N=1)* | 2.9 [2.1, 4.7] | 3.4 [2.4, 5.3] | 0.047 |
| Preoperative CEA (median [IQR]) | *Missing (N=18)* | 12.2 [3.6, 51.2] | 21.0 [7.0, 93.0] | 0.008 |
| Bilobar (%) | Unilobar | 55 (50) | 94 (37) | 0.015 |
|  | Bilobar | 54 (50) | 162 (63) |  |
| Extrahepatic disease (%) | No | 94 (86) | 213 (83) | 0.468 |
|  | Yes | 15 (14) | 43 (17) |  |
| Resection margin (%) | R0 | 97 (90) | 200 (78) | 0.009 |
|  | R1 | 11 (10) | 56 (22) |  |
|  | *Missing (N=1)* |  |  |  |
| CRS (%) | Low (0-2) | 48 (48) | 89 (37) | 0.073 |
|  | High (3-5) | 53 (52) | 151 (63) |  |
|  | *Incomplete (N=24)* |  |  |  |
| Major resection (≥3 complete segments) (%) | No major resection | 67 (61) | 129 (50) | 0.052 |
|  | Major resection | 42 (39) | 127 (50) |  |
| Major complications (i.e. Clavien-Dindo ≥3) | No | 99 (91) | 228 (89) | 0.614 |
|  | Yes | 10 (9) | 28 (11) |  |
| Postoperative death (%) | No | 109 (100) | 249 (97) | 0.081 |
|  | Yes | 0 (0) | 7 (3) |  |
