# Supplementary material for: Angiogenic desmoplastic histopathological growth pattern as a prognostic marker of good outcome in patients with colorectal liver metastases
Source: Angiogenesis. 2019 Jan 12;22(2):355–68. doi: 10.1007/s10456-019-09661-5 (PMC6475515; doi:10.1007/s10456-019-09661-5)
Supplement: Supplementary file 3 — Supplementary table 3. Baseline characteristics chemo-naive versus pre-treated patients (DOCX 19 KB) [file 10456_2019_9661_MOESM3_ESM.docx]

| **Supplementary table 3. Baseline characteristics chemo-naive versus pre-treated patients** | | | | | |
| --- | --- | --- | --- | --- | --- |
|  |  | **Total**  **N=732** | **Chemo-naive**  **N=367 (50%)** | **Pre-treated**  **N=365 (50%)** | **P- value** |
| Gender | Male | 469 (64%) | 233 (64%) | 236 (65%) | 0.742 |
|  | Female | 263 (36%) | 134 (37%) | 129 (35%) |  |
|  |  |  |  |  |  |
| Age | Median (IQR) | 64 (58-71) | 66 (59-73) | 63 (56-69) | <0.001* |
|  |  |  |  |  |  |
| ASA | ASA I-II | 656 (91%) | 322 (90%) | 334 (92%) | 0.398 |
|  | ASA > II | 66 (9%) | 36 (10 %) | 30 (8%) |  |
|  | *Missing* | *10 patients* |  |  |  |
|  |  |  |  |  |  |
| **Primary tumour characteristics** |  |  |  |  |  |
| Location | Right-sided | 120 (16%) | 62 (17%) | 58 (16 %) | 0.194 |
|  | Left-sided | 307 (42%) | 147 (40%) | 160 (44%) |  |
|  | Rectum | 289 (40%) | 146 (40%) | 143 (39%) |  |
|  | Double tumour | 16 (2%) | 12 (3%) | 4 (1%) |  |
|  |  |  |  |  |  |
| pTumour stage | pT0-2 | 136 (20%) | 80 (22%) | 56 (17%) | 0.082 |
|  | pT3-4 | 562 (81%) | 284 (78.0%) | 278 (83.2%) |  |
|  | *Missing* | *34 patients* |  |  |  |
|  |  |  |  |  |  |
| Nodal status | N0 | 274 (40%) | 153 (42%) | 121 (36%) | 0.104 |
|  | N+ | 420 (61%) | 208 (58%) | 212 (64%) |  |
|  | *Missing* | *38 patients* |  |  |  |
|  |  |  |  |  |  |
| Adjuvant chemotherapy | No | 623 (86%) | 290 (79%) | 333 (92%) | <0.001* |
|  | Yes | 105 (14%) | 77 (21%) | 28 (8%) |  |
|  | *Missing* | *4 patients* |  |  |  |
|  |  |  |  |  |  |
| **CRLM characteristics** |  |  |  |  |  |
| Synchronous CRLM | No | 337 (46%) | 255 (70%) | 82 (23%) | <0.001* |
|  | Yes | 395 (54%) | 112 (31%) | 283 (78%) |  |
|  |  |  |  |  |  |
| Disease-free interval | Median (IQR) | 1 (0-17) | 13 (0-25) | 0 (0-2) | <0.001* |
|  |  |  |  |  |  |
| Number of CRLM | Median (IQR) | 2 (1-4) | 1 (1-2) | 3 (2-5) | <0.001* |
|  |  |  |  |  |  |
| Size of largest CRLM | Median (IQR) | 3.1 (2.1-4.7) | 3.0 (3.0-4.0) | 3.2 (2.3-5.2) | 0.002* |
|  | *Missing* | *2 patients* |  |  |  |
|  |  |  |  |  |  |
| Preoperative CEA | Median (IQR) | 14.7 (4.8-51.8) | 11.0 (4.2-29.8) | 19.7 (5.3-74.0) | <0.001* |
|  | *Missing* | *32 patients* |  |  |  |
|  |  |  |  |  |  |
| Fong CRS | Low | 420 (60%) | 283 (79%) | 137 (40%) | <0.001* |
|  | High | 280 (40%) | 76 (21%) | 204 (60%) |  |
|  | *Incomplete CRS* | *32 patients* |  |  |  |
|  |  |  |  |  |  |
| Bilobar metastases | No | 435 (59%) | 286 (78%) | 149 (41%) | <0.001* |
|  | Yes | 297 (41%) | 81 (22%) | 216 (59%) |  |
|  |  |  |  |  |  |
| Resection margin | R0 | 621 (85%) | 324 (89%) | 297 (82%) | 0.006* |
|  | R1 | 108 (15%) | 41 (11%) | 67 (18%) |  |
|  | *Missing* | *3 patients* |  |  |  |
|  |  |  |  |  |  |
| HGP type | Desmoplastic | 177 (24%) | 68 (19%) | 109 (30%) | <0.001* |
|  | Replacement | 86 (12%) | 73 (20%) | 13 (4%) |  |
|  | Mixed | 469 (64%) | 226 (62%) | 243 (67%) |  |
|  |  |  |  |  |  |
| Extra Hepatic Disease | No | 648 (89%) | 341 (93%) | 307 (84%) | <0.001* |
|  | Yes | 84 (12%) | 26 (7%) | 58 (16%) |  |
|  |  |  |  |  |  |
| Major liver resection | <3 complete segments | 481 (66%) | 285 (78%) | 196 (54%) | <0.001* |
|  | ≥3 complete segments | 251 (34%) | 82 (22%) | 169 (46%) |  |
|  |  |  |  |  |  |
| Major complications | No | 665 (91%) | 338 (92%) | 327 (90%) | 0.193 |
|  | Yes | 66 (9%) | 28 (8%) | 38 (10%) |  |
|  | *Missing* | *1 patient* |  |  |  |
|  |  |  |  |  |  |
| Postoperative death | No | 718 (98%) | 360 (98%) | 358 (98%) | 0.992 |
|  | Yes | 14 (2%) | 7 (2%) | 7 (2%) |  |
| Percentages do not always add up to 100% due to rounding. Abbreviations in alphabetical order: ASA: American Society of Anaesthesiologists; CEA: carcinoembryonic antigen; CRLM: colorectal liver metastases; CRS: clinical risk score; HGP: histopathological growth pattern; IQR: interquartile range; R1: irradical resection margin | | | | | |
