# Supplementary material for: Angiogenic desmoplastic histopathological growth pattern as a prognostic marker of good outcome in patients with colorectal liver metastases
Source: Angiogenesis. 2019 Jan 12;22(2):355–68. doi: 10.1007/s10456-019-09661-5 (PMC6475515; doi:10.1007/s10456-019-09661-5)
Supplement: Supplementary file 4 — Supplementary table 4. Uni- and multivariable logistic regression analysis for association with dHGP (DOCX 14 KB) [file 10456_2019_9661_MOESM4_ESM.docx]

| **Supplementary table 4. Uni- and multivariable logistic regression analysis for association with dHGP** | | | | |
| --- | --- | --- | --- | --- |
|  | **Univariable** | | **Multivariable** |  |
| **Variable** | **Odds Ratio [95% CI]** | **P-value** | **Odds Ratio [95% CI]** | **P-value** |
| Right-sided primary | 1.112 [0.710-1.742] | 0.644 | 1.264 [0.789-2.026] | 0.330 |
| pT3-4 | 0.786 [0.517-1.196] | 0.261 | 0.849 [0.534-1.351] | 0.491 |
| Node positive primary | 0.702 [0.495-0.995] | 0.047* | 0.611 [0.415-0.901] | 0.013* |
| Disease free interval (cont.) | 0.989 [0.978-1.000] | 0.049* | 0.992 [0.980-1.005] | 0.227 |
| Number of CRLM (cont.) | 0.977 [0.909-1.050] | 0.530 | 0.872 [0.790-0.962] | 0.006* |
| Diameter largest CRLM (cont.) | 0.904 [0.832-0.982] | 0.017* | 0.898 [0.822-0.981] | 0.017* |
| Preoperative CEA level (cont.) | 1.000 [0.999-1.000] | 0.800 | 1.000 [0.999-1.001] | 0.932 |
| Preoperative chemotherapy | 1.872 [1.325-2.646] | <0.001* | 2.709 [1.746-4.203] | <0.001* |
| Abbreviations in alphabetical order: CEA: carcinoembryonic antigen; CI: confidence interval; cont.: continuous CRLM: colorectal liver metastases; dHGP: desmoplastic histopathological growth pattern | | | | |
