# Supplementary material for: Angiogenic desmoplastic histopathological growth pattern as a prognostic marker of good outcome in patients with colorectal liver metastases
Source: Angiogenesis. 2019 Jan 12;22(2):355–68. doi: 10.1007/s10456-019-09661-5 (PMC6475515; doi:10.1007/s10456-019-09661-5)
Supplement: Supplementary file 6 — Supplementary table 6. Overall Survival Cox regression analysis all neoadjuvantly treated patients +/- Bevacizumab (DOCX 14 KB) [file 10456_2019_9661_MOESM6_ESM.docx]

| **Supplementary table 6. Cox regression all neoadjuvantly treated patients +/- Bevacizumab** | | | | |
| --- | --- | --- | --- | --- |
| **Overall Survival** | **Univariable** | | **Multivariable** | |
| **Variable** | **Hazard Ratio [95% CI]** | **P-value** | **Hazard Ratio [95% CI]** | **P-value** |
| Age at resection CRLM (cont.) | 1.021 [1.007-1.036] | 0.003 | 1.034 [1.016-1.052] | <0.001 |
| ASA > II | 1.082 [0.675-1.733] | 0.744 | 1.195 [0.726-1.967] | 0.484 |
| Right-sided primary | 0.877 [0.590-1.304] | 0.517 | 0.952 [0.623-1.456] | 0.821 |
| pT3-4 | 1.476 [0.988-2.204] | 0.057 | 1.398 [0.896-2.182] | 0.140 |
| Node positive primary | 1.419 [1.050-1.918] | 0.023 | 1.382 [0.990-1.928] | 0.057 |
| Disease free interval (cont.) | 0.996 [0.985-1.008] | 0.541 | 0.996 [0.983-1.009] | 0.532 |
| Number of CRLM (cont.) | 1.023 [0.976-1.072] | 0.340 | 1.052 [0.995-1.112] | 0.074 |
| Diameter largest CRLM (cont.) | 0.997 [0.952-1.045] | 0.905 | 1.025 [0.968-1.086] | 0.394 |
| Preoperative CEA level (cont.) | 1.000 [1.000-1.000] | 0.955 | 1.000 [1.000-1.000] | 0.558 |
| R1 resection CRLM | 1.374 [0.989-1.908] | 0.058 | 1.274 [0.868-1.872] | 0.216 |
| Extra hepatic disease | 1.705 [1.222-2.380] | 0.002 | 1.725 [1.164-2.558] | 0.007 |
| dHGP | 0.661 [0.484-0.902] | 0.009 | 0.906 [0.635-1.293] | 0.587 |
| Bevacizumab | 1.001 [0.758-1.324] | 0.992 | 1.063 [0.777-1.456] | 0.702 |
