# Supplementary material for: Angiogenic desmoplastic histopathological growth pattern as a prognostic marker of good outcome in patients with colorectal liver metastases
Source: Angiogenesis. 2019 Jan 12;22(2):355–68. doi: 10.1007/s10456-019-09661-5 (PMC6475515; doi:10.1007/s10456-019-09661-5)
Supplement: Supplementary file 7 — Supplementary table 7. Progression-Free Survival Cox regression all neoadjuvantly treated patients +/- Bevacizumab (DOCX 14 KB) [file 10456_2019_9661_MOESM7_ESM.docx]

| **Supplementary table 7. Cox regression all neoadjuvantly treated patients +/- Bevacizumab** | | | | |
| --- | --- | --- | --- | --- |
| **Progression Free Survival** | **Univariable** | | **Multivariable** | |
| **Variable** | **Hazard Ratio [95% CI]** | **P-value** | **Hazard Ratio [95% CI]** | **P-value** |
| Age at resection CRLM (cont.) | 1.008 [0.996-1.019] | 0.188 | 1.012 [0.998-1.026] | 0.091 |
| ASA > II | 1.086 [0.731-1.614] | 0.682 | 1.048 [0.683-1.608] | 0.830 |
| Right-sided primary | 0.936 [0.684-1.282] | 0.681 | 1.046 [0.747-1.466] | 0.791 |
| pT3-4 | 1.420 [1.021-1.974] | 0.037 | 1.442 [1.004-2.070] | 0.047 |
| Node positive primary | 1.328 [1.032-1.710] | 0.028 | 1.143 [0.867-1.507] | 0.343 |
| Disease free interval (cont.) | 0.994 [0.985-1.004] | 0.234 | 0.997 [0.986-1.008] | 0.578 |
| Number of CRLM (cont.) | 1.026 [0.989-1.063] | 0.174 | 1.036 [0.993-1.082] | 0.103 |
| Diameter largest CRLM (cont.) | 0.993 [0.954-1.034] | 0.728 | 1.000 [0.954-1.048] | 0.989 |
| Preoperative CEA level (cont.) | 1.000 [1.000-1.000] | 0.462 | 1.000 [1.000-1.000] | 0.489 |
| R1 resection CRLM | 1.464 [1.101-1.948] | 0.009 | 1.456 [1.046-2.026] | 0.026 |
| Extra hepatic disease | 1.777 [1.321-2.390] | <0.001 | 1.872 [1.336-2.625] | <0.001 |
| dHGP | 0.671 [0.519-0.867] | 0.002 | 0.752 [0.562-1.007] | 0.055 |
| Bevacizumab | 0.986 [0.776-1.253] | 0.908 | 1.087 [0.833-1.419] | 0.540 |
