# Supplementary material for: Angiogenic desmoplastic histopathological growth pattern as a prognostic marker of good outcome in patients with colorectal liver metastases
Source: Angiogenesis. 2019 Jan 12;22(2):355–68. doi: 10.1007/s10456-019-09661-5 (PMC6475515; doi:10.1007/s10456-019-09661-5)
Supplement: Supplementary file 8 — Supplementary table 8. Baseline characteristics chemo-naive patients 50% cut-off (DOCX 18 KB) [file 10456_2019_9661_MOESM8_ESM.docx]

| **Supplementary table 8. Baseline characteristics chemo-naive patients 50% cut-off** | | | | | | |
| --- | --- | --- | --- | --- | --- | --- |
|  |  | **>50% dHGP** | **>50% rHGP** | **>50% pHGP** | | **p-value** |
|  |  | **142 (39%)** | **213 (58%)** | **8 (2%)** | |  |
| **General characteristics** |  |  |  |  | |  |
| Age at resection (median [IQR]) |  | 68.0 [58.2, 76.0] | 65.0 [60.0, 72.0] | 68.0 [55.5, 73.2] | | 0.320 |
| Gender (%) | Female | 46 (32) | 80 (38) | 4 (50) | | 0.426 |
|  | Male | 96 (68) | 133 (62) | 4 (50) | |  |
| ASA classification(%) | ASA Class I-II | 122 (87) | 188 (91) | 8 (100) | | 0.290 |
|  | ASA Class >II | 18 (13) | 18 (9) | 0 (0) | |  |
|  | *Missing (N=9)* |  |  |  | |  |
| **Primary tumour characteristics** |  |  |  |  | |  |
| Location (%) | Right-sided | 23 (16) | 37 (17) | 1 (12) | | 0.700 |
|  | Left-sided | 62 (44) | 82 (38) | 2 (25) | |  |
|  | Rectum | 53 (37) | 87 (41) | 4 (50) | |  |
|  | Double tumour | 4 (3) | 7 (3) | 1 (12) | |  |
| Pathological T-stage (%) | pT 0-2 | 36 (25) | 40 (19) | 3 (38) | | 0.210 |
|  | pT 3-4 | 106 (75) | 170 (81) | 5 (62) | |  |
|  | *Missing (N=3)* |  |  |  | |  |
| Pathological N-stage (%) | N0 | 75 (53) | 75 (36) | 2 (25) | | 0.004* |
|  | N+ | 66 (47) | 133 (64) | 6 (75) | |  |
|  | *Missing (N=6)* |  |  |  | |  |
| Adjuvant chemotherapy (%) | No | 120 (85) | 161 (76) | 7 (88) | | 0.107 |
|  | Yes | 22 (15) | 52 (24) | 1 (12) | |  |
| **CRLM characteristics** |  |  |  |  | |  |
| Synchronous CRLM (%) | Metachronous | 95 (67) | 152 (71) | 4 (50) | | 0.333 |
|  | Synchronous | 47 (33) | 61 (29) | 4 (50) | |  |
| DFI (median [IQR]) |  | 13.0 [0.0, 26.0] | 13.0 [0.0, 24.0] | 2.0 [0.0, 6.5] | | 0.044 |
| Number of CRLM (median [IQR]) |  | 1.0 [1.0, 2.0] | 1.0 [1.0, 2.0] | 1.0 [1.0, 1.0] | | 0.271 |
| Largest diameter CRLM (median [IQR]) | *Missing (N=1)* | 2.8 [1.9, 4.0] | 3.0 [2.0, 4.0] | 4.0 [2.9, 4.9] | | 0.066 |
| Preoperative CEA (median [IQR]) | *Missing (N=14)* | 7.8 [3.4, 17.5] | 13.7 [5.0, 51.5] | 20.6 [2.9, 23.3] | | 0.002* |
| Bilobar (%) | Unilobar | 110 (77) | 167 (78) | 7 (88) | | 0.796 |
|  | Bilobar | 32 (23) | 46 (22) | 1 (12) | |  |
| Extrahepatic disease (%) | No | 136 (96) | 194 (91) | 8 (100) | | 0.171 |
|  | Yes | 6 (4) | 19 (9) | 0 (0) | |  |
| Resection margin (%) | R0 | 133 (94) | 181 (86) | 8 (100) | | 0.040* |
|  | R1 | 9 (6) | 30 (14) | 0 (0) | |  |
|  | *Missing (N=2)* |  |  |  | |  |
| CRS (%) | Low (0-2) | 112 (81) | 162 (78) | 6 (75) | | 0.692 |
|  | High (3-5) | 26 (19) | 47 (22) | 2 (25) | |  |
|  | *Incomplete (N=8)* |  |  |  | |  |
| Major resection (≥3 complete segments) (%) | No major resection | 118 (83) | 158 (74) | 7 (88) | 0.112 | |
|  | Major resection | 24 (17) | 55 (26) | 1 (12) |  | |
| Major complications (i.e. Clavien-Dindo ≥3) | No | 127 (89) | 199 (94) | 8 (100) | 0.220 | |
|  | Yes | 15 (11) | 13 (6) | 0 (0) |  | |
|  | *Missing (N=1)* |  |  |  |  | |
| Postoperative death (%) | No | 140 (99) | 208 (98) | 8 (100) | 0.757 | |
|  | Yes | 2 (1) | 5 (2) | 0 (0) |  | |
