# Supplementary material for: Angiogenic desmoplastic histopathological growth pattern as a prognostic marker of good outcome in patients with colorectal liver metastases
Source: Angiogenesis. 2019 Jan 12;22(2):355–68. doi: 10.1007/s10456-019-09661-5 (PMC6475515; doi:10.1007/s10456-019-09661-5)
Supplement: Supplementary file 9 — Supplementary table 9. Uni- and multivariable Cox regression analysis OS of chemo-naive patients >50% cut-off (DOCX 15 KB) [file 10456_2019_9661_MOESM9_ESM.docx]

| **Supplementary table 9. Uni- and multivariable Cox regression analysis OS of chemo-naive patients >50% cut-off** | | | | | |
| --- | --- | --- | --- | --- | --- |
| **Overall Survival** | | **Univariable** | | **Multivariable** | |
| **Variable** | | **Hazard Ratio [95% CI]** | **P-value** | **Hazard Ratio [95% CI]** | **P-value** |
| Age at resection CRLM (cont.) | | 1.012 [0.998-1.026] | 0.100 | 1.016 [1.001-1.032] | 0.038 |
| ASA > II | | 1.021 [0.650-1.606] | 0.927 | 1.047 [0.650-1.685] | 0.850 |
| Right-sided primary | | 1.472 [1.046-2.073] | 0.027 | 1.540 [1.066-2.224] | 0.021 |
| pT3-4 | | 1.142 [0.816-1.599] | 0.438 | 0.868 [0.604-1.248] | 0.446 |
| Node positive primary | | 1.421 [1.072-1.883] | 0.015 | 1.473 [1.068-2.030] | 0.018 |
| Disease free interval (cont.) | | 0.998 [0.991-1.005] | 0.535 | 0.991 [0.984-0.999] | 0.022 |
| Number of CRLM (cont.) | | 1.138 [1.022-1.266] | 0.018 | 1.164 [1.025-1.322] | 0.019 |
| Diameter largest CRLM (cont.) | | 1.096 [1.037-1.159] | 0.001 | 1.124 [1.044-1.209] | 0.002 |
| Preoperative CEA level (cont.) | | 1.001 [1.001-1.002] | 0.002 | 1.001 [1.000-1.002] | 0.155 |
| R1 resection CRLM | | 1.278 [0.852-1.915] | 0.236 | 1.063 [0.689-1.640] | 0.781 |
| Extra hepatic disease | | 1.490 [0.879-2.526] | 0.139 | 1.719 [0.925-3.196] | 0.087 |
| dHGP | | Ref |  | Ref |  |
|  | rHGP | 2.154 [1.581-2.935] | <0.001 | 1.917 [1.367-2.688] | <0.001 |
|  | pHGP | 5.073 [2.113-12.177] | <0.001 | 4.398 [1.829-10.577] | <0.001 |
