# Supplementary material for: Angiogenic desmoplastic histopathological growth pattern as a prognostic marker of good outcome in patients with colorectal liver metastases
Source: Angiogenesis. 2019 Jan 12;22(2):355–68. doi: 10.1007/s10456-019-09661-5 (PMC6475515; doi:10.1007/s10456-019-09661-5)
Supplement: Supplementary file 10 — Supplementary table 10. Uni- and multivariable logistic regression analysis for association with dHGP >50% cut-off (DOCX 14 KB) [file 10456_2019_9661_MOESM10_ESM.docx]

| **Supplementary table 10. Uni- and multivariable logistic regression analysis for association with dHGP >50% cut-off** | | | | |
| --- | --- | --- | --- | --- |
|  | **Univariable** | | **Multivariable** |  |
| **Variable** | **Odds Ratio [95% CI]** | **P-value** | **Odds Ratio [95% CI]** | **P-value** |
| Right-sided primary | 1.030 [0.691-1.533] | 0.886 | 1.046 [0.676-1.617] | 0.841 |
| pT3-4 | 0.709 [0.480-1.047] | 0.084 | 0.750 [0.482-1.166] | 0.201 |
| Node positive primary | 0.564 [0.411-0.773] | <0.001* | 0.499 [0.348-0.715] | <0.001* |
| Disease free interval (cont.) | 0.988 [0.979-0.997] | 0.006* | 0.995 [0.985-1.005] | 0.326 |
| Number of CRLM (cont.) | 1.093 [1.023-1.168] | 0.008* | 0.965 [0.891-1.046] | 0.390 |
| Diameter largest CRLM (cont.) | 1.058 [0.991-1.129] | 0.089 | 1.055 [0.977-1.139] | 0.175 |
| Preoperative CEA level (cont.) | 1.000 [0.999-1.000] | 0.726 | 0.999 [0.999-1.000] | 0.056 |
| Preoperative chemotherapy | 3.228 [2.370-4.395] | <0.001* | 4.052 [2.708-6.063] | <0.001* |
| Abbreviations in alphabetical order: CEA: carcinoembryonic antigen; CI: confidence interval; cont.: continuous CRLM: colorectal liver metastases; dHGP: desmoplastic histopathological growth pattern | | | | |
