# Supplementary material for: Angiogenic desmoplastic histopathological growth pattern as a prognostic marker of good outcome in patients with colorectal liver metastases
Source: Angiogenesis. 2019 Jan 12;22(2):355–68. doi: 10.1007/s10456-019-09661-5 (PMC6475515; doi:10.1007/s10456-019-09661-5)
Supplement: Supplementary file 11 — Supplementary table 11. Baseline characteristics pre-treated patients >50% cut-off (DOCX 18 KB) [file 10456_2019_9661_MOESM11_ESM.docx]

| **Supplementary table 11. Baseline characteristics pre-treated patients >50% cut-off** | | | | | |
| --- | --- | --- | --- | --- | --- |
|  |  | **>50% dHGP** | **>50% rHGP** | **>50% pHGP** | **p-value** |
|  |  | **N=241 (67%)** | **N=112 (31%)** | **N=5 (1%)** |  |
| **General characteristics** |  |  |  |  |  |
| Age at resection (median [IQR]) |  | 63.0 [56.0, 70.0] | 64.0 [57.8, 69.0] | 62.0 [60.0, 64.0] | 0.422 |
| Gender (%) | Female | 84 (35) | 42 (38) | 1 (20) | 0.682 |
|  | Male | 157 (65) | 70 (62) | 4 (80) |  |
| ASA classification(%) | ASA Class I-II | 219 (91) | 103 (92) | 5 (100) | 0.773 |
|  | ASA Class >II | 21 (9) | 9 (8) | 0 (0) |  |
|  | *Missing (N=1)* |  |  |  |  |
| **Primary tumour characteristics** |  |  |  |  |  |
| Location (%) | Right-sided | 41 (17) | 16 (14) | 0 (0) | 0.637 |
|  | Left-sided | 100 (41) | 54 (48) | 4 (80) |  |
|  | Rectum | 97 (40) | 41 (37) | 1 (20) |  |
|  | Double tumour | 3 (1) | 1 (1) | 0 (0) |  |
| Pathological T-stage (%) | pT 0-2 | 44 (20) | 11 (11) | 1 (20) | 0.174 |
|  | pT 3-4 | 181 (80) | 88 (89) | 4 (80) |  |
|  | *Missing (N=31)* |  |  |  |  |
| Pathological N-stage (%) | N0 | 93 (41) | 24 (24) | 2 (40) | 0.015 |
|  | N+ | 132 (59) | 74 (76) | 3 (60) |  |
|  | *Missing (N=32)* |  |  |  |  |
| Adjuvant chemotherapy (%) | No | 224 (94) | 100 (90) | 4 (80) | 0.282 |
|  | Yes | 15 (6) | 11 (10) | 1 (20) |  |
|  | *Missing (N=4)* |  |  |  |  |
| **CRLM characteristics** |  |  |  |  |  |
| Synchronous CRLM (%) | Metachronous | 58 (24) | 22 (20) | 1 (20) | 0.646 |
|  | Synchronous | 183 (76) | 90 (80) | 4 (80) |  |
| DFI (median [IQR]) |  | 0.0 [0.0, 3.0] | 0.0 [0.0, 0.0] | 0.0 [0.0, 0.0] | 0.608 |
| Number of CRLM (median [IQR]) |  | 3.0 [2.0, 5.0] | 3.0 [2.0, 5.2] | 6.0 [2.0, 10.0] | 0.322 |
| Largest diameter CRLM (median [IQR]) | *Missing (N=1)* | 3.3 [2.3, 5.1] | 3.1 [2.0, 5.2] | 5.1 [2.5, 5.4] | 0.619 |
| Preoperative CEA (median [IQR]) | *Missing (N=18)* | 19.7 [5.2, 65.2] | 21.0 [6.8, 112.5] | 3.0 [2.4, 21.0] | 0.170 |
| Bilobar (%) | Unilobar | 101 (42) | 44 (39) | 2 (40) | 0.896 |
|  | Bilobar | 140 (58) | 68 (61) | 3 (60) |  |
| Extrahepatic disease (%) | No | 203 (84) | 95 (85) | 5 (100) | 0.625 |
|  | Yes | 38 (16) | 17 (15) | 0 (0) |  |
| Resection margin (%) | R0 | 199 (83) | 87 (78) | 5 (100) | 0.281 |
|  | R1 | 41 (17) | 25 (22) | 0 (0) |  |
|  | *Missing (N=1)* |  |  |  |  |
| CRS (%) | Low (0-2) | 102 (45) | 32 (31) | 1 (20) | 0.047 |
|  | High (3-5) | 126 (55) | 70 (69) | 4 (80) |  |
|  | *Incomplete (N=24)* |  |  |  |  |
| Major resection (≥3 complete segments) (%) | No major resection | 139 (58) | 52 (46) | 2 (40) | 0.117 |
|  | Major resection | 102 (42) | 60 (54) | 3 (60) |  |
| Major complications (i.e. Clavien-Dindo ≥3) | No | 218 (90) | 97 (87) | 5 (100) | 0.407 |
|  | Yes | 23 (10) | 15 (13) | 0 (0) |  |
| Postoperative death (%) | No | 238 (99) | 108 (96) | 5 (100) | 0.323 |
|  | Yes | 3 (1) | 4 (4) | 0 (0) |  |
