# Supplementary material for: Angiogenic desmoplastic histopathological growth pattern as a prognostic marker of good outcome in patients with colorectal liver metastases
Source: Angiogenesis. 2019 Jan 12;22(2):355–68. doi: 10.1007/s10456-019-09661-5 (PMC6475515; doi:10.1007/s10456-019-09661-5)
Supplement: Supplementary file 12 — Supplementary table 12. Uni- and multivariable Cox regression analysis for OS of pre-treated patients >50% cut-off (DOCX 15 KB) [file 10456_2019_9661_MOESM12_ESM.docx]

| **Supplementary table 12. Uni- and multivariable Cox regression analysis for OS of pre-treated patients >50% cut-off** | | | | | |
| --- | --- | --- | --- | --- | --- |
| **Overall Survival** | | **Univariable** | | **Multivariable** | |
| **Variable** | | **Hazard Ratio [95% CI]** | **P-value** | **Hazard Ratio [95% CI]** | **P-value** |
| Age at resection CRLM (cont.) | | 1.021 [1.007-1.036] | 0.004 | 1.032 [1.015-1.050] | <0.001 |
| ASA > II | | 1.089 [0.680-1.746] | 0.722 | 1.218 [0.741-2.001] | 0.436 |
| Right-sided primary | | 0.919 [0.618-1.369] | 0.679 | 0.988 [0.646-1.511] | 0.954 |
| pT3-4 | | 1.476 [0.988-2.206] | 0.057 | 1.341 [0.860-2.092] | 0.196 |
| Node positive primary | | 1.466 [1.081-1.989] | 0.014 | 1.411 [1.010-1.972] | 0.044 |
| Disease free interval (cont.) | | 0.997 [0.986-1.009] | 0.640 | 0.995 [0.983-1.008] | 0.448 |
| Number of CRLM (cont.) | | 1.024 [0.977-1.073] | 0.324 | 1.058 [1.000-1.121] | 0.051 |
| Diameter largest CRLM (cont.) | | 0.994 [0.949-1.043] | 0.817 | 1.031 [0.973-1.093] | 0.299 |
| Preoperative CEA level (cont.) | | 1.000 [1.000-1.000] | 0.938 | 1.000 [1.000-1.000] | 0.504 |
| R1 resection CRLM | | 1.364 [0.979-1.902] | 0.067 | 1.246 [0.851-1.825] | 0.258 |
| Extra hepatic disease | | 1.746 [1.243-2.454] | 0.001 | 1.815 [1.221-2.698] | 0.003 |
| dHGP | | Ref |  | Ref |  |
|  | rHGP | 1.570 [1.183-2.084] | 0.002 | 1.282 [0.922-1.784] | 0.140 |
|  | pHGP | 1.020 [0.324-3.209] | 0.973 | 0.829 [0.245-2.801] | 0.763 |
