# Supplementary figures and images for: Angiogenic desmoplastic histopathological growth pattern as a prognostic marker of good outcome in patients with colorectal liver metastases
Source: Angiogenesis. 2019 Jan 12;22(2):355–68. doi: 10.1007/s10456-019-09661-5 (PMC6475515; doi:10.1007/s10456-019-09661-5)

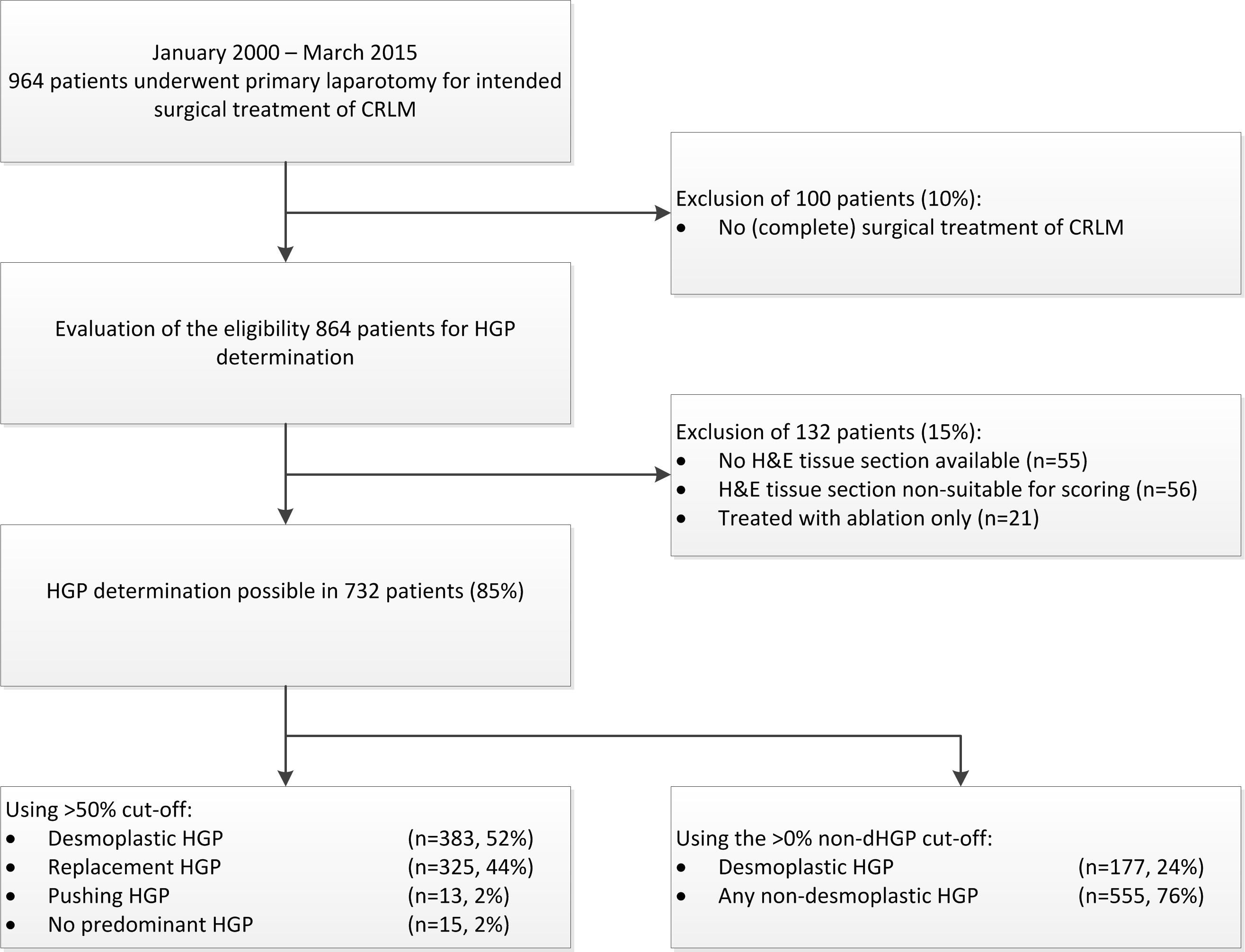

Supplement: Supplementary file 13 — Supplementary figure 1. A flowchart of the patient inclusion (TIF 15495 KB) [file 10456_2019_9661_MOESM13_ESM.tif]

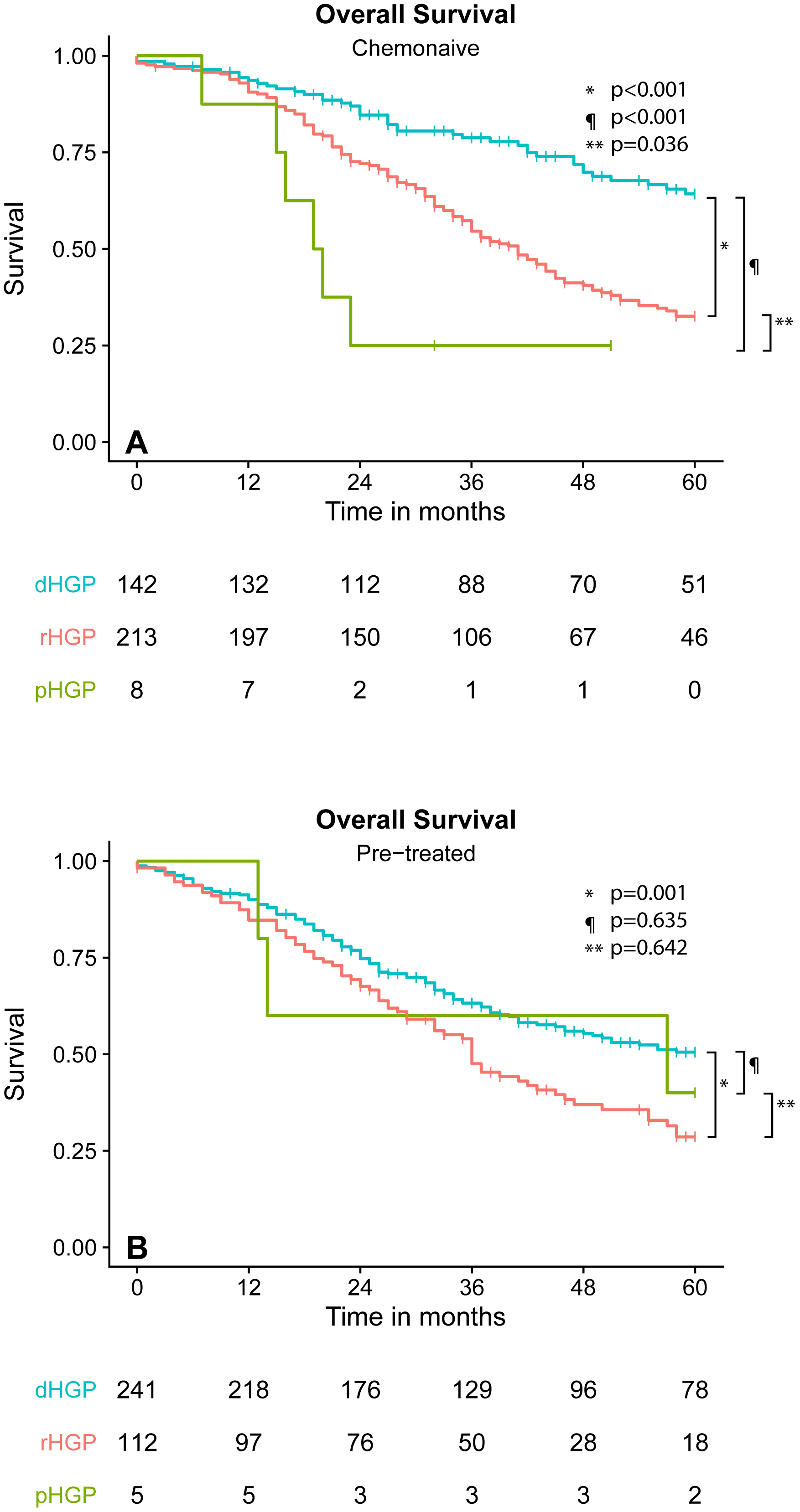

Supplement: Supplementary file 14 — Supplementary figure 2A-B. OS using the >50% cut-off. 2A: OS chemo-naive patients. 2B: OS pre-treated patients (TIF 1177 KB) [file 10456_2019_9661_MOESM14_ESM.tif]
