# Supplementary material for: Angiogenic desmoplastic histopathological growth pattern as a prognostic marker of good outcome in patients with colorectal liver metastases
Source: Angiogenesis. 2019 Jan 12;22(2):355–68. doi: 10.1007/s10456-019-09661-5 (PMC6475515; doi:10.1007/s10456-019-09661-5)
Supplement: Supplementary file 5 — Supplementary table 5. (Rebuttal table 2.) Uni- and multivariable logistic regression analysis for association with dHGP in the neoadjuvantly treated group (DOCX 14 KB) [file 10456_2019_9661_MOESM5_ESM.docx]

| **Supplementary table 5. Uni- and multivariable logistic regression analysis for association with dHGP in the neoadjuvantly treated group** | | | | |
| --- | --- | --- | --- | --- |
|  | **Univariable** | | **Multivariable** |  |
| **Variable** | **Odds Ratio [95% CI]** | **P-value** | **Odds Ratio [95% CI]** | **P-value** |
| Right-sided primary | 1.289 [0.711-2.337] | 0.403 | 1.421 [0.757-2.670] | 0.274 |
| pT3-4 | 0.806 [0.441-1.473] | 0.484 | 0.805 [0.417-1.552] | 0.517 |
| Node positive primary | 0.707 [0.440-1.137] | 0.152 | 0.655 [0.388-1.107] | 0.114 |
| Disease free interval (cont.) | 1.005 [0.988-1.024] | 0.556 | 1.001 [0.980-1.023] | 0.926 |
| Number of CRLM (cont.) | 0.906 [0.829-0.991] | 0.032 | 0.891 [0.803-0.989] | 0.030 |
| Diameter largest CRLM (cont.) | 0.933 [0.850-1.024] | 0.145 | 0.936 [0.847-1.035] | 0.200 |
| Preoperative CEA level (cont.) | 1.000 [0.999-1.000] | 0.728 | 1.000 [0.999-1.001] | 0.912 |
| Bevacizumab | 1.449 [0.906-2.317] | 0.121 | 1.595 [0.951-2.675] | 0.077 |
